# Synthetic Tailoring of Graphene Nanostructures with Zigzag‐Edged Topologies: Progress and Perspectives

**DOI:** 10.1002/anie.202008838

**Published:** 2020-10-06

**Authors:** Junzhi Liu, Xinliang Feng

**Affiliations:** ^1^ Department of Chemistry and State Key Laboratory of Synthetic Chemistry The University of Hong Kong Pokfulam Road Hong Kong China; ^2^ Center for Advancing Electronics Dresden (cfaed) & Faculty of Chemistry and Food Chemistry Technische Universität Dresden 01062 Dresden Germany

**Keywords:** bottom-up synthesis, doping, graphene nanoribbons, magnetism, nanographenes

## Abstract

Experimental and theoretical investigations have revealed that the chemical and physical properties of graphene are crucially determined by their topological structures. Therefore, the atomically precise synthesis of graphene nanostructures is essential. A particular example is graphene nanostructures with zigzag‐edged structures, which exhibit unique (opto)electronic and magnetic properties owing to their spin‐polarized edge state. Recent progress in the development of synthetic methods and strategies as well as characterization methods has given access to this class of unprecedented graphene nanostructures, which used to be purely molecular objectives in theoretical chemistry. Thus, clear insight into the structure–property relationships has become possible as well as new applications in organic carbon‐based electronic and spintronic devices. In this Minireview, we discuss the recent progress in the controlled synthesis of zigzag‐edged graphene nanostructures with different topologies through a bottom‐up synthetic strategy.

## Graphical abstract

This Minireview summarizes the design, synthesis, and exotic properties of graphene nanostructures with dominant zigzag‐edged topologies. Deep insights into the advantages and challenges of their synthetic methods and strategies are provided to encourage future endeavors and developments.

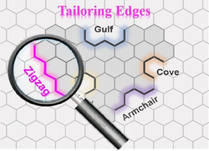

## 1 Introduction

In 2004, Novoselov, Geim et al. isolated the first stable graphene as single‐ to few‐layer hexagonal carbon nanosheets by using a micromechanical cleavage method.^[1]^ This groundbreaking experiment stimulated research studies in graphene and carbon nanostructures. The outstanding electronic and optical properties of graphene not only constitute an important issue in fundamental physics but also hold promise for use in future nanoelectronics.[^2^, ^3^, ^4^, ^5^] Nonetheless, the conduction and valence bands of graphene cross at the Dirac points, thereby leading to a zero‐band gap semiconductor, which dramatically limits the integration of graphene into digital electronic devices.^[6]^ Therefore, finding a way to open the band gap in graphene is of great importance. Many top‐down approaches involving band‐gap opening, such as substrate‐induced band‐gap tuning,[^7^, ^8^] bilayer graphene,^[9]^ hydrogen passivation,^[10]^ or nanoscale holes to create graphene nanomeshes^[11]^ have been reported. The most prominent method is to realize quantum confinement of charge carriers with tailorable band gaps in graphene nanostructures by cutting graphene into finite graphene fragments and strips, so‐called nanographenes (NGs) and graphene nanoribbons (GNRs).[^12^, ^13^, ^14^, ^15^, ^16^, ^17^]

**Figure 1:**
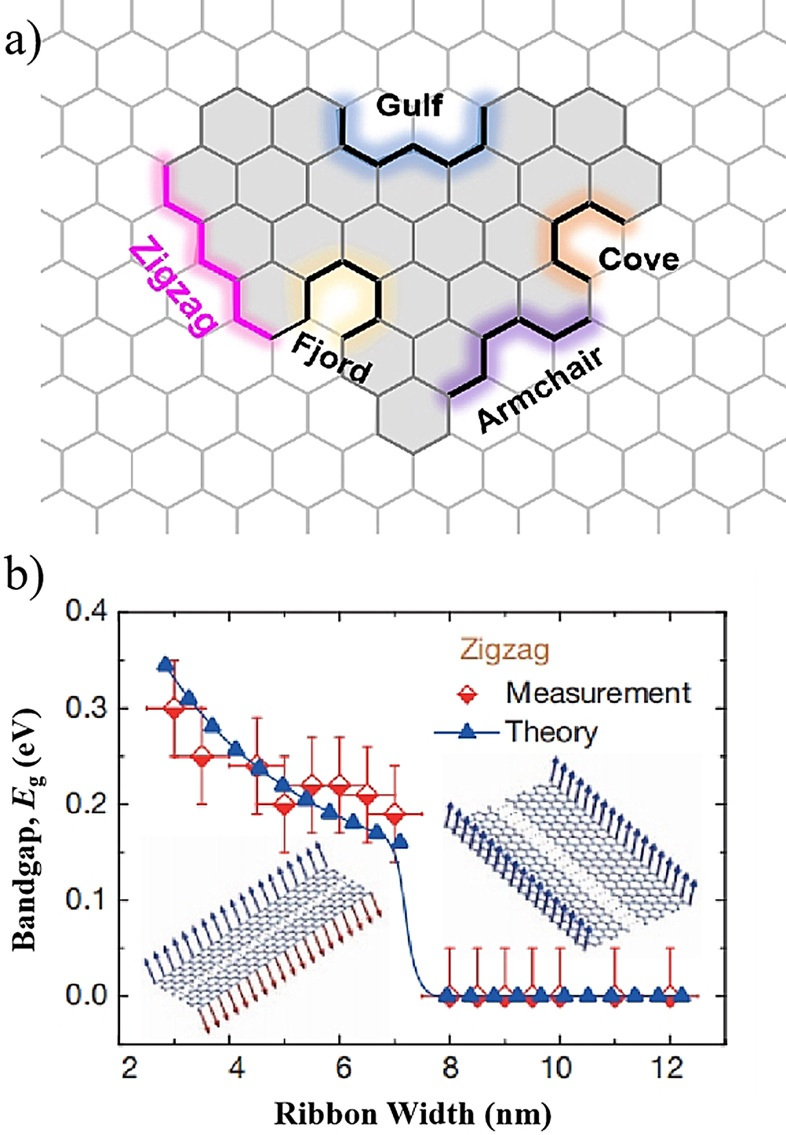
a) Different edge structures of graphene. b) In ZGNRs, the edge magnetism can be tailored by the ribbon width.^[24]^

According to theoretical calculations, the magnetic and electronic properties of NGs and GNRs, as well as their chemical reactivities, are crucially determined by their edge types.[^18^, ^19^] In general, there are five types of edge structures for graphene: zigzag, armchair, cove, gulf, and fjord (Figure 1 a). In contrast to armchair‐, cove‐, gulf‐, and fjord‐edged graphene nanostructures, which generally present semiconducting properties, most zigzag‐edged NGs exhibit distinct magnetic features because of the spin polarization associated with their edge states.[^20^, ^21^] For example, Enoki and co‐workers experimentally confirmed the edge state of zigzag‐edged hydrogen‐terminated NGs by using scanning tunneling microscopy (STM) and scanning tunneling spectroscopy (STS).[^22^, ^23^] Tapasztó and co‐workers demonstrated spin ordering along the edges in narrow zigzag‐edged graphene nanoribbons (ZGNRs; Figure 1 b). The ferromagnetic and antiferromagnetic coupling with the zigzag and armchair edges as well as their switching behavior were elucidated.^[24]^ Although substantial efforts have been dedicated in the last decade to preparing high‐quality NGs, the above top‐down approaches suffer from difficulties in controlling the sizes and edge structures.^[25]^ For example, the top‐down‐fabricated narrow GNRs show ruffled edges, which make the band gap poorly defined, thereby resulting in dramatically degraded charge‐carrier transport properties.^[26]^ This constitutes the main reason why achieving NGs with atomically smooth edges has been a major obstacle for applying graphene in nanoelectronic devices.

**Figure 2:**
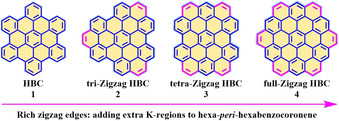
The chemical structures of representative zigzag‐edged HBCs.

**Figure 3:**
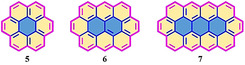
Circumacene‐based NGs with full zigzag‐edged structures.

In contrast with the top‐down method, the bottom‐up synthetic strategy has the incomparable advantage of controlling the widths and edge topologies of NGs at the atomic level.[^3^, ^14^, ^27^] The classical bottom‐up synthesis of NGs or large polycyclic aromatic hydrocarbons (PAHs) involves a typical oxidative intramolecular cyclodehydrogenation of dendritic oligophenylene precursors. By using such a synthetic strategy, a broad class of PAHs with different topological structures have been synthesized in recent decades.[^3^, ^28^] In contrast to the armchair‐edged NGs (A‐NGs) with a fully benzenoid structure, zigzag‐edged NGs (Z‐NGs) have another ring fused at the bay position (pink; Figure 2), and the two additional π‐electrons cannot be drawn as a Clar sextet.^[29]^ For example, the fully benzenoid hexa‐*peri*‐hexabenzocoronene (HBC, **1**) can be annulated with three, four, or six additional benzene rings at the bay regions to generate a tri‐zigzag HBC (**2**),^[30]^ tetra‐zigzag HBC (**3**),^[31]^ and full‐zigzag HBC (**4**; also called supercoronene), respectively (Figure 2). Such a zigzag K‐region has double‐bond character, thus enabling its electrophilic substitution reaction or oxidation to the diketone structure.^[32]^ Despite the failure in the synthesis of full zigzag HBC (**4**) thus far, another family of PAHs with a full zigzag periphery, namely, circumacenes, has received much synthetic success (Figure 3). Among these, coronene (**5**) and ovalene (**6**) date back to efforts from Clar in the last century.[^33^, ^34^, ^35^, ^36^] In 1991, Broene and Diederich described a synthetic route for the next generation circumarenes, namely, circumanthracene (**7**; Figure 3).^[37]^ However, they could not characterize this compound because of its poor solubility. Even three decades after its discovery, there have been no significant efforts towards the synthesis and application of circumanthracene, its derivatives, or the next generation of circumacenes.

**Figure 4:**
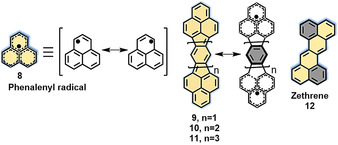
Phenalenyl radical (**8**) and its resonance forms, biphenalenyls **9**–**11** and their resonance forms, and zethrene (**12**).

The integration of zigzag edges or reactive double bonds into NGs exerts a large influence on the electronic and magnetic properties, as well as their chemical reactivity, such as in oxidation reactions or electrophilic substitutions. For example, our group demonstrated an edge chlorination method for the functionalization of coronene, whereby the chlorine atoms at the edges provide the opportunity for further chemical derivatization to produce a thiol‐substituted coronene.[^32^, ^38^] On the other hand, zigzag‐edged NGs generally have non‐Kekulé structures or open‐shell structures because of the unpaired electrons present in the molecules,[^39^, ^40^] which render them ideal candidates for application in nanocarbon‐based spintronics. A prominent example is phenalenyl radical[^41^, ^42^] (**8**; Figure 4) with a delocalized spin/radical structure. In the first study of **8** in the 1950s,[^43^, ^44^] it was found to be very reactive and only survived under an inert gas atmosphere in solution. Later, **8** was stabilized by protection with three *tert*‐butyl groups, which allowed its isolation and characterization.[^45^, ^46^] Since the 1990s, phenalenyl radicals have been widely explored as building blocks for constructing open‐shell polycyclic hydrocarbons (PHs), and Nakasuji and Kubo have intensively investigated a series of biphenalenyl derivatives (**9**–**11**; Figure 4).[^47^, ^48^] Another outstanding molecule rich in zigzag edges is zethrene (**12**; Figure 4), a unique PAH with formally fixed C−C double bonds. Clar first synthesized zethrene (**12**) in 1955.^[49]^ Later, in the 1960s, Staab and Sondheimer developed more straightforward synthetic methods through the cross‐coupling of copper acetylides and iodoarenes.[^50^, ^51^, ^52^] Recently, the groups of Tobe and Wu have paid particular attention to zethrene and its higher homologues (such as heptazethrene and octazethrene) with open‐shell characteristics.[^53^, ^54^, ^55^, ^56^, ^57^] Very recently, we in collaboration with the Fasel group demonstrated the on‐surface synthesis of super‐heptazethrene on Au(111).^[58]^ In contrast to its open‐shell singlet ground state in solution, super‐heptazethrene presents a closed‐shell character on Au(111). Since there have already been excellent reviews summarizing phenalenyl‐based[^47^, ^48^] and zethrene‐type radicals,[^59^, ^60^, ^61^, ^62^, ^63^] we will not discuss them further in this Minireview.

In the last decade, a remarkable breakthrough was achieved in the bottom‐up synthesis of zigzag‐edged NGs and GNRs, which benefited from the advancement of synthetic methods and analytical tools. In particular, powerful and complementary on‐surface and in‐solution synthetic strategies have been established; in the former case, the characterization of NGs at the atomic/molecular level is currently possible.[^17^, ^64^] For example, open‐shell *peri*‐tetracene[^65^, ^66^] and *peri*‐pentacene,^[67]^ the next highest analogues of bisanthene, which have been pursued for several decades but hampered by their poor chemical stabilities, are now accessible by solution‐based or surface‐assisted syntheses. π‐Extended triangulene^[68]^ with three unpaired electrons and chemically unstable full zigzag‐edged GNRs^[69]^ have also been recently realized by on‐surface synthesis. All of these graphene nanostructures show a critical dependence of their electronic properties on the topological structures, especially with the dominant zigzag edges.

In this Minireview, we will highlight recent advances in the synthetic strategies and physiochemical properties of graphene nanostructures rich in zigzag‐edges, including *peri*‐acenes, triangular‐shaped NGs, rhombus NGs, heteroatom‐doped NGs, and zigzag‐edged GNRs. We will provide our views on the advantages and challenges of the respective synthetic methods and routes. Moreover, the unique chemical, electronic, photophysical, and magnetic properties of these graphene nanostructures will be discussed in this context.

## 2 peri‐Acenes

**Figure 5:**
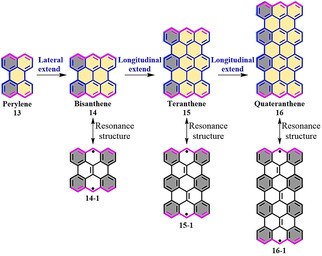
Chemical structures of zigzag‐edged NGs based on bisanthene.

Acenes are linearly *cata*‐condensed PAHs, while *peri*‐acenes consist of two or more rows of *peri*‐fused acenes (Figure 5).^[70]^ Generally, the solution synthesis of longer acenes (larger than hexacenes) remains elusive because of their poor stability under ambient conditions,^[71]^ whereas higher acenes up to dodecacene have been achieved through on‐surface synthesis.[^72^, ^73^, ^74^, ^75^] Bisanthene (**14**) can be regarded as a laterally extended perylene (**13**; Figure 5), and was synthesized for the first time in 1948.[^76^, ^77^] Recently, homologous PAHs with zigzag‐edge peripheries, such as teranthene (**15**) and quateranthene (**16**),^[40]^ were synthesized by expanding the bisanthene core in the longitudinal direction (Figure 5). From the resonance structures, there are three and four additional Clar sextets for teranthene (**15‐1**) and quateranthene (**16‐1**), respectively. According to the Clar sextet rule, more sextets in the open‐shell biradical form results in higher aromatic stabilization energies and thus a more dominant contribution of the biradical form to the ground state (Figure 5).^[40]^ From calculations, the biradical indexes (*y*_0_) of **15** and **16** are estimated to be 0.42 (CASSCF) and 0.84 (UBHandHLYP), respectively, in contrast to only 0.07 (CASSCF) for **14**. The open‐shell characteristics of **15** and **16** also lead to their unique optical and electronic properties.^[78]^ For example, **15** and **16** display weak low‐energy absorption at *λ*=1054 nm (**15**) and *λ*=1147 nm (**16**) in the NIR region, which indicates that the energy gap between their highest occupied molecular orbital (HOMO) and lowest unoccupied molecular orbital (LUMO) is small.

**Figure 6:**
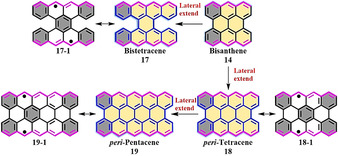
Chemical structures of the zigzag‐edged NGs based on bisanthene.

**Scheme 1:**
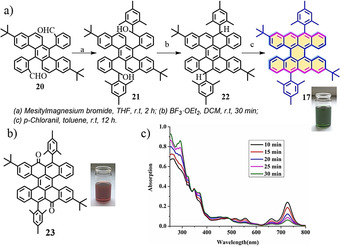
a) Synthesis of bistetracene **17**. b) Structural formula of **23**. c) Time‐dependent UV/Vis spectra of **17** under ambient conditions.^[81]^

According to theoretical calculations, the energy gap of *peri*‐acenes drastically decreases as their length increases.[^79^, ^80^] In 2015, we reported the synthesis of bistetracene **17** (tetrabenzo[*a*,*f*,*j*,*o*]perylene),^[81]^ in which two tetracenes are fused together by two bonds (Figure 6). First, **20** was synthesized in eight steps. Then, bistetracene **17** was synthesized through a Grignard reaction, ring fusion, and oxidative dehydrogenation (Scheme 1 a). The optical energy gap (*E*_g_^opt^) of **17** was estimated to be 1.56 eV from its UV/Vis absorption spectrum. Interestingly, based on the calculation, **17** has a biradical nature (*y*_0_=0.61) in the ground state. The driving force to form biradical **17‐1** is due to the three additional Clar sextets in the open‐shell form compared to its closed‐shell form (Figure 6). However, **17** is unstable under ambient conditions, with a half‐life (*t*_1/2_) of only about 30 min (Scheme 1 c). A red‐colored solution was obtained during the oxidation, which corresponds to the formation of tetrabenzo[*a*,*f*,*j*,*o*]perylene‐9,19‐dione (**23**; Scheme 1 b).

**Scheme 2:**
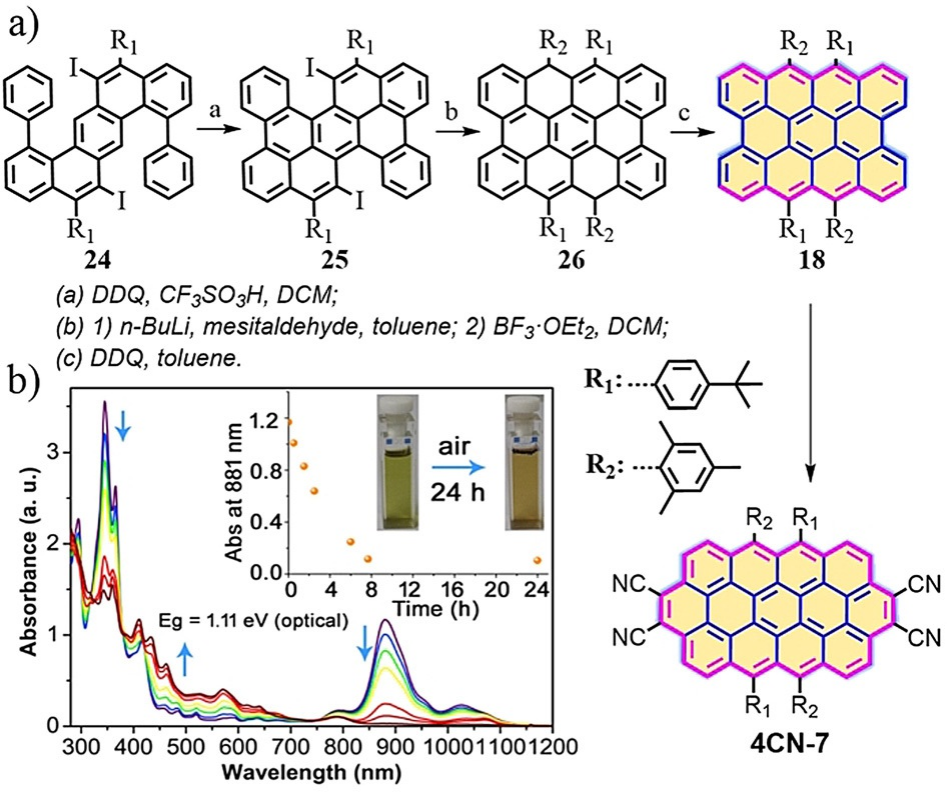
a) Synthetic route towards *peri*‐tetracene **18** and **4CN‐7** through Diels–Alder reactions of **18**. b) Time‐dependent UV/Vis absorption spectra of **18**.^[65]^

In 2018, the next highest analogue, *peri*‐tetracene **18**, was synthesized in solution by us and by Wu (Scheme 2).[^65^, ^66^] The synthesis of *peri*‐tetracene **18** was achieved by dehydrogenation of **26** with 2,3‐dichloro‐5,6‐dicyano‐1,4‐benzoquinone (DDQ, Scheme 2). The UV/Vis absorption spectrum of **18** shows an intense absorption centered at *λ*=881 nm along with two shoulders (*λ*=788 and 1021 nm) in the NIR region (Scheme 2 b). Accordingly, the optical energy gap (*E*_g_^opt^) of **18** was estimated to be 1.11 eV, which is much smaller than that of bistetracene **17** (1.56 eV). Moreover, from the DFT calculations (UHF/6‐31G*), the singlet biradical character (*y*_0_) of *peri*‐tetracene **18** is 0.72, which is higher than that of **17** (*y*_0_=0.61). Similar to **17**, *peri*‐tetracene **18** is also unstable under ambient conditions, with a half‐life (*t*_1/2_) estimated to be about 3 h (Scheme 2 b). Moreover, we also demonstrated the surface‐assisted synthesis of unsubstituted *peri*‐tetracene via the key monomer 7,14‐di(2‐methylphenyl)benzo[*k*]tetraphene.^[82]^ Remarkably, the solution‐based synthesis of *peri*‐tetracene **18** enabled the in situ generation of circumanthracene derivatives.[^65^, ^83^] For example, the novel circumanthracene derivative **4CN**‐**7** with four cyano groups was produced by two Diels–Alder (DA) reactions at the bay position of *peri*‐tetracene **18** by using DDQ (Scheme 2 a). This reaction can be attributed to the relatively low‐lying LUMO (−3.50 eV) of **18**.

**Figure 7:**
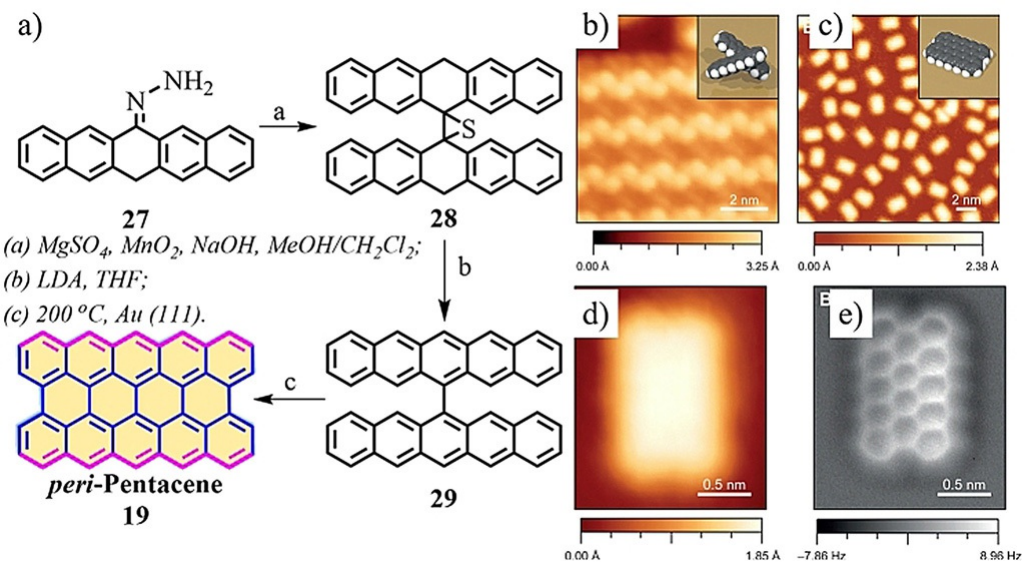
a) Synthesis of **19** on a gold surface. b) STM image of **29**. c,d) STM images of **19**. e) nc‐AFM image of **19**.^[67]^

Researchers have not abandoned the pursuit of higher *peri*‐acenes, even though their synthesis has been met with tremendous challenges. Thus far, two synthetic approaches have been proposed for the synthesis of *peri*‐pentacene (**19**) in solution; unfortunately, both were unsuccessful.[^84^, ^85^] In 2015, *peri*‐pentacene (**19**) was reported through on‐surface synthesis from the precursor 6,6′‐bipentacene (**29**) under ultrahigh vacuum (UHV) conditions (Figure 7).^[67]^ After depositing **29** on the Au(111) surface, it arranged into highly ordered linear chains, as visualized by STM imaging (Figure 7 b). Annealing at 200 °C, led to **29** being fully cyclized to form *peri*‐pentacene (**19**), as confirmed by STM and noncontact‐AFM (nc‐AFM) with a CO‐functionalized tip (Figure 7 c–e). Interestingly, the highly reactive **19** could be stabilized on the surface through interaction with the free valences of the Au substrate.

## 3 Triangular‐Shaped Nanographenes

**Figure 8:**
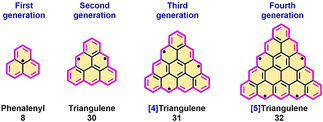
The nomenclature of higher triangular‐shaped nanographenes.

**Scheme 3:**
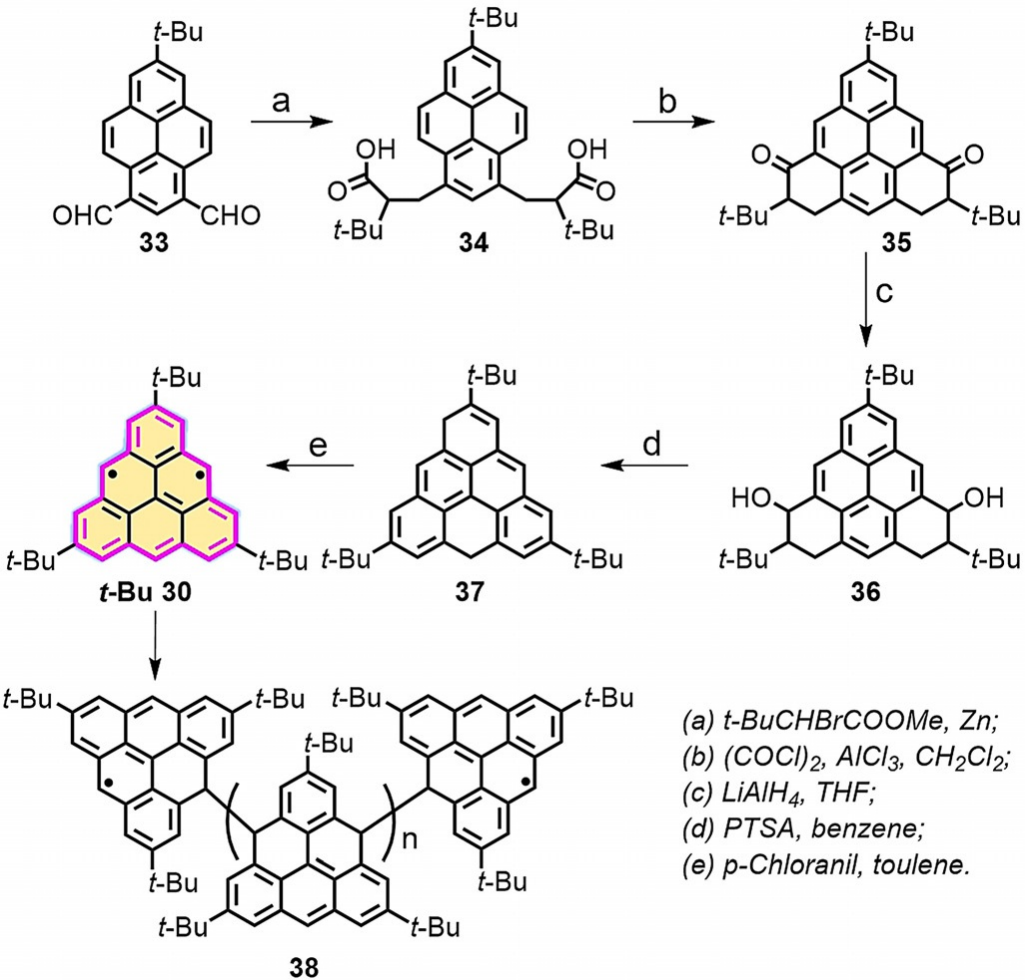
Synthesis of triangulene ***t*** **‐Bu 30** with three *tert*‐butyl groups.^[91]^

*C*_3_‐Symmetric triangulene (**30**) was first studied by Clar in 1941,^[86]^ and with two unpaired electrons is one of the most fundamental non‐Kekulé PAHs (Figure 8).^[87]^ Moreover, the singlet–triplet energy gap (Δ*E*_S‐T_) of triangulene (**30**) was calculated to be 20 kcal mol^−1^, thus suggesting that **30** displays a triplet biradical feature in the ground state.^[88]^ Clar and Stewart made the first attempt to synthesize **30**; however, only the polymerized compound was obtained, which indicates the thermodynamic instability of **30**.[^89^, ^90^] In 2001, Morita, Nakasuji, and co‐workers introduced *tert*‐butyl (*t*‐Bu) groups onto the zigzag edges of triangulene to kinetically protect the reactive edges (***t*** **‐Bu 30**; Scheme 3).^[91]^ However, this compound was only confirmed by electron‐spin resonance (ESR). In addition, this *tert*‐butyl‐protected triangulene was not stable in solution, with oligomers forming even at room temperature (Scheme 3).

**Figure 9:**
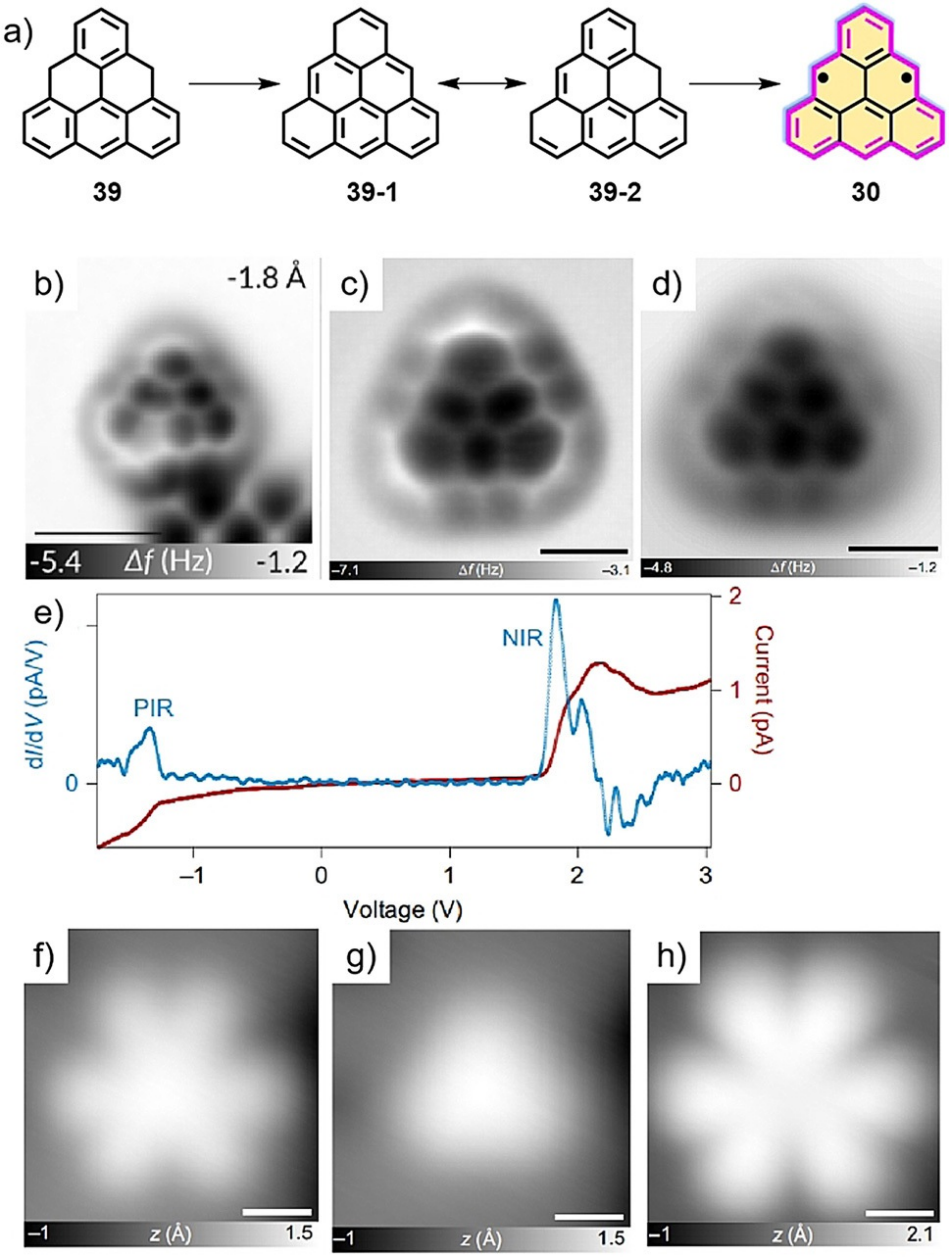
a) Synthesis of triangulene **30** on a surface. b) AFM images of **30** on NaCl. c) AFM images of **30** on Cu. d) AFM images of **30** on Xe. e) The differential conductance *dI*/*dV*(V) of **30**. f‐h) STM images of **30** at different voltages (f: *V=*−1.4 V; g: *V=*0.1 V; h:*V=*1.85 V).^[92]^

In 2017, Pavliček, Gross et al. demonstrated the surface‐assisted synthesis of triangulene (**30**) under UHV conditions,^[92]^ whereby dihydrotriangulenes (**39**; Figure 9 a) were deposited on NaCl(100), Cu(111), and Xe(111) surfaces and **30** formed by atomic manipulation (Figure 9 b–d). The differential conductance d*I*/d*V*(V) of **30** was measured (Figure 9 e). There are two clear peaks at *V=*−1.4 V and *V=*1.85 V, which correspond to the negative and positive ion resonances. Figure 9 f–h shows the STM images of **30** at different voltages, with the image taken in the gap region (*V=*0.1 V) appearing to be triangular‐shaped. Very recently, Frederiksen and co‐workers reported the on‐surface synthesis of graphene flakes based on the [3]triangulene molecules, which possess a spin *S*=1 ground state.^[93]^

**Figure 10:**
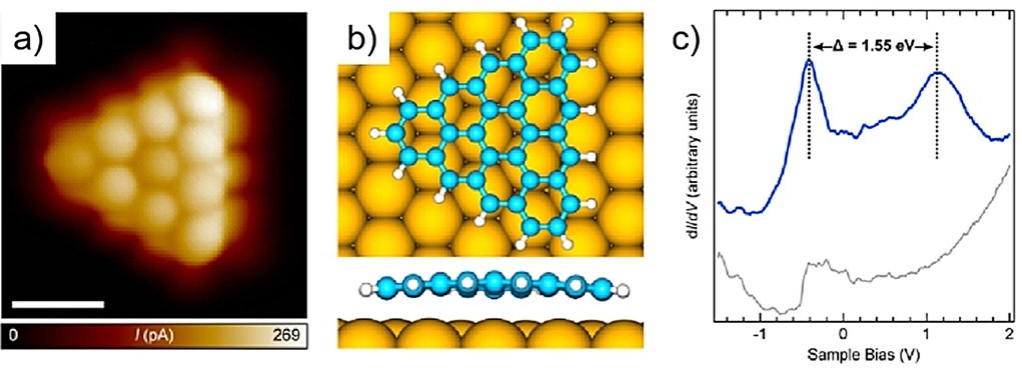
a) STM image of [4]triangulene (**31**). b) DFT‐simulated geometry of **31** on the Au(111) surface. c) dI/dV spectrum of **31** (blue curve).^[68]^

**Scheme 4:**
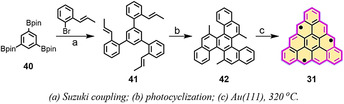
Synthetic route towards [4]triangulene **31**.^[68]^

The next generation in the family of PAHs with triangular topology, [4]triangulene (**31,** C_33_H_15_) with three unpaired electrons, was recently reported by the Fasel group and us (Scheme 4).^[68]^ The key precursor **42** with three strategically installed methyl substituents was achieved through a photocyclization reaction of **41**. Subsequently, **42** was sublimated on the Au(111) surface, and then ring‐closure reactions of the methyl groups were performed by annealing at 320 °C to afford **31**. The structure of **31** was confirmed by ultrahigh‐resolution STM (Figure 10 a), which shows **31** adopts a planar structure on the surface through adsorption onto Au(111), which is also in line with the DFT calculations (Figure 10 b). The electronic properties of **31** were studied by STS (Figure 10 c), and the electronic band of **31** was estimated to be 1.55 eV. Moreover, calculations based on density functional, tight‐binding, and many‐body perturbation theory were carried out, which indicated that [4]triangulene (**31**) maintains an open‐shell quartet ground state on the surface.

**Figure 11:**
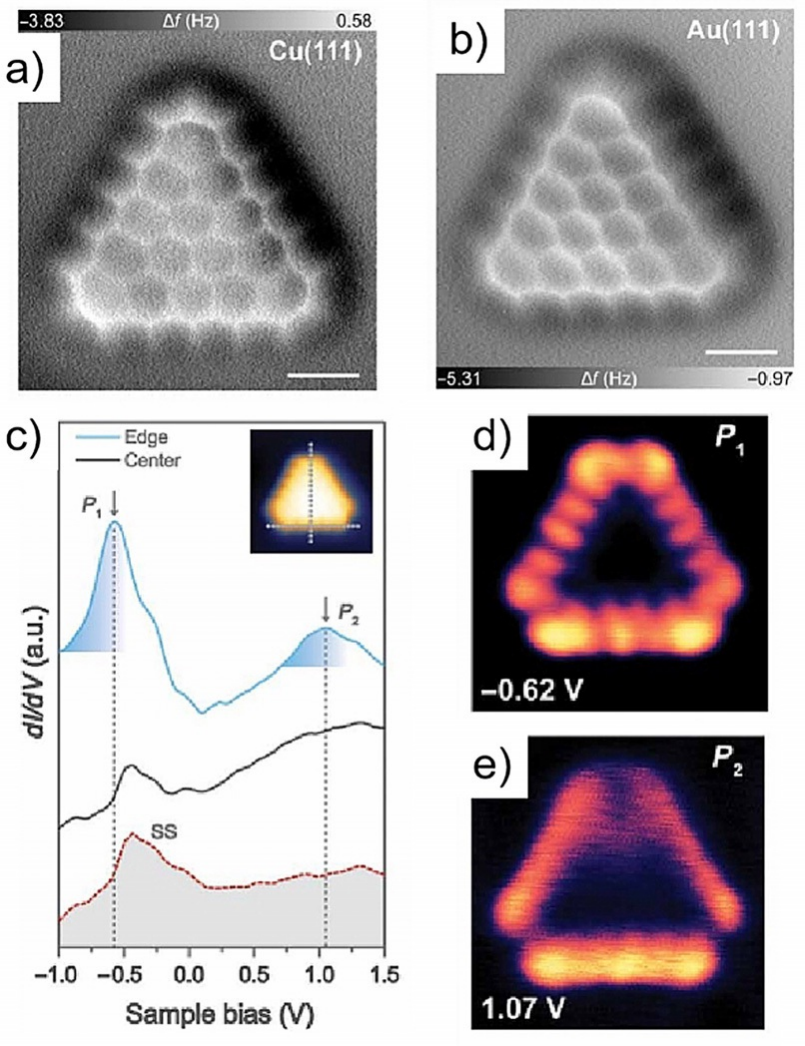
Structural characterization of **32** on a) Cu(111) and b) Au(111) surfaces. c) d*I*/d*V* spectra of **32** (solid blue line). d,e) Experimental d*I*/d*V* maps performed at different energy positions (d: −0.62 V; e: 1.07 V).^[94]^

**Scheme 5:**
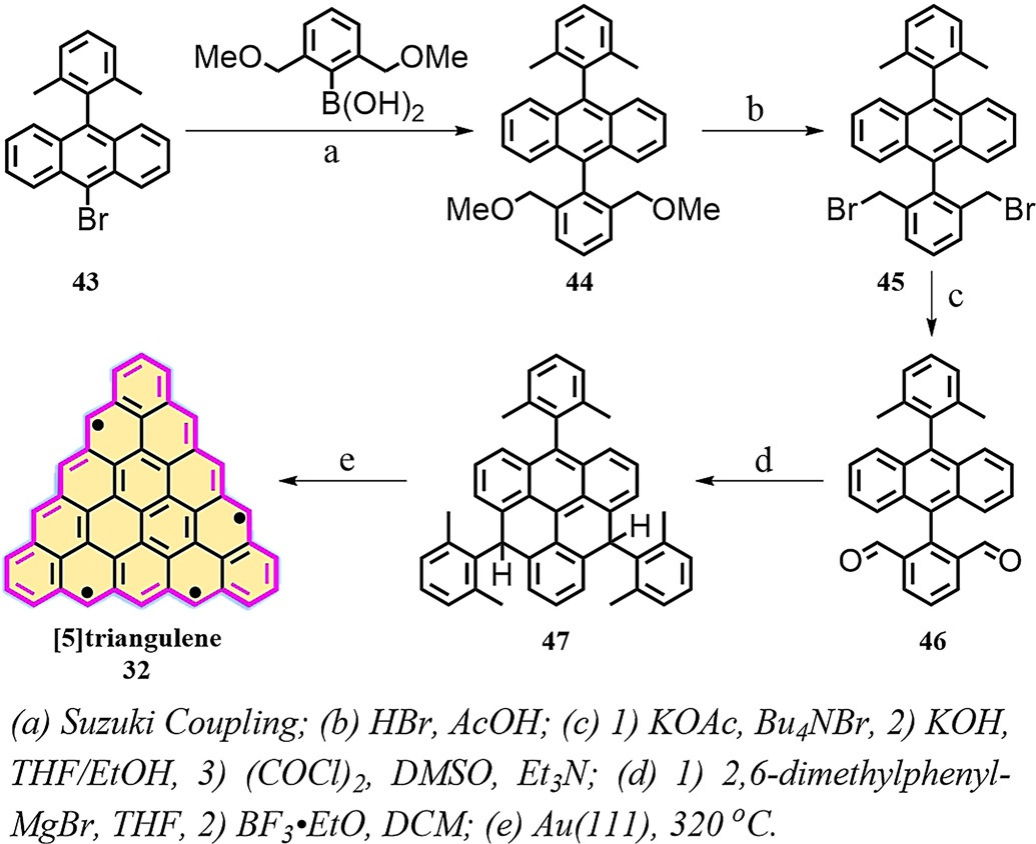
The synthetic route towards [5]triangulene **32**.^[94]^

In 2019, Wu et al. demonstrated the synthesis of the next‐generation triangulene, [5]triangulene (**32**), by surface‐assisted cyclodehydrogenation (Scheme 5).^[94]^ Precursor **47** was sublimated onto Cu(111) (Figure 11 a) and Au(111) (Figure 11 b) surfaces. The STM study showed the successful formation of [5]triangulene **32** with the expected *D*_3*h*_ symmetry. Interestingly, the yield on Cu(111) (ca. 60 %, ca. 500 K) was much higher than that on the Au(111) substrate (ca. 5 %, ca. 600 K). The STS measurements to unveil the electronic properties were performed on Au(111) substrates, since they possess weak interactions with [5]triangulene (Figure 11 c). Conductance (d*I*/d*V*) mapping of the [5]triangulene molecule was conducted using a metallic tip, in which the *P*_1_ state revealed five bright spots located at the zigzag edge of **32** (Figure 11 d), while the *P*_2_ state exhibited an edge‐localized pattern (Figure 11 e).

**Figure 12:**
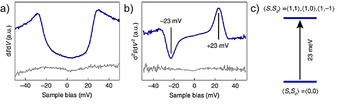
a) d*I*/d*V* and b) d^2^ *I*/d*V* ^2^ spectra of **53**. c) Spin excitation of **53**. *S*_z_ denotes the spin projection quantum number.^[99]^

**Figure 13:**
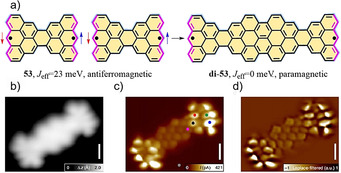
a) The formation of **di‐53** by the linear fusion of **53**. b) High‐resolution STM image of **di‐53**. c,d) Ultrahigh‐resolution STM images of **di‐53**.^[99]^

**Scheme 6:**
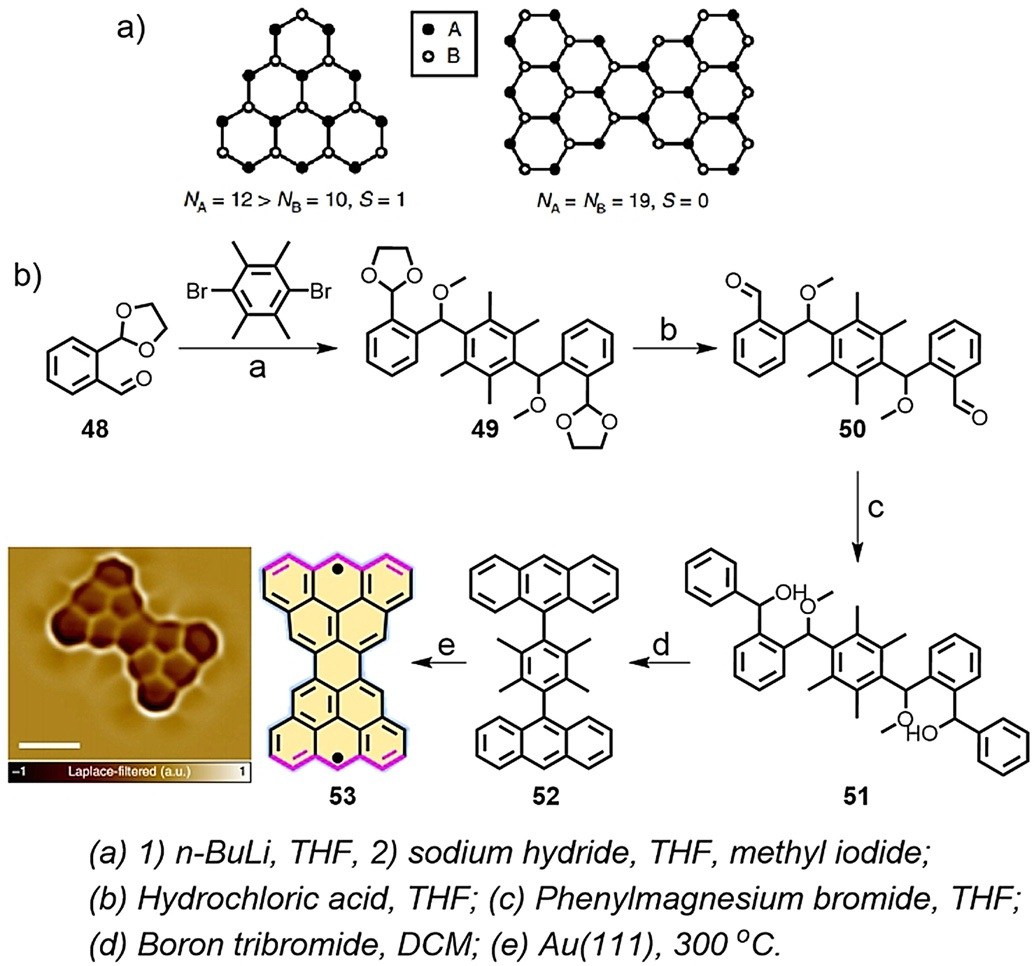
a) Schematic illustration of the difference between white and black (or unstarred and starred) vertices for triangulene and Clar's goblet **53**. b) The synthetic route towards bowtie‐shaped nanographene **53**. Insert: ultrahigh‐resolution STM image of **53**.^[99]^

In 1972, Clar and MacKay predicted the bowtie‐shaped PAH **53** (C_38_H_18_; Scheme 6),^[95]^ in which two [3]triangulenes are connected head‐to‐head. It is important to emphasize that **53** belongs to the family of the as then undiscovered concealed non‐Kekulé structures.[^96^, ^97^] A concealed non‐Kekulé molecule displays no Kekulé structure and possesses the same number of white and black (or unstarred and starred) vertices (Scheme 6 a).^[98]^ Like triangulene, a Kekulé structure of **53** cannot be drawn with paired electrons. Very recently, we in collaboration with the Fasel group demonstrated the first synthesis of Clar's goblet PAH **53** through combined in‐solution and on‐surface synthesis.^[99]^ The synthesis of **53** involves the key precursor **52** (Scheme 6 b), in which the four methyl groups serve as ring‐closure units. The generation of **52** involved a multistep synthesis (Scheme 6 b). Precursor **52** was deposited onto Au(111) and annealed at 300 °C to boost the on‐surface synthesis. The successful formation of **53** was confirmed by ultrahigh‐resolution STM (inset Figure in Scheme 6 b), with the two radicals located at the terminal zigzag edges. Moreover, STM and spin excitation spectroscopic analysis of **53** on the Au(111) surface showed it had a strong antiferromagnetic character with an exchange coupling of 23 meV (an effective exchange parameter *J*_eff_=23 meV; Figure 12), which is larger than that of the Landauer limit of minimum energy dissipation at room temperature. Interestingly, this electronic decoupling was confirmed when **53** linearly fused to form its dimer structure (**di‐53**; Figure 13 a). This arises because the electron spins in **di‐53** are separated by a large nonmagnetic unit. The chemical structure of **di‐53** was clearly confirmed by ultrahigh‐resolution STM (Figure 13 b–d).

## 4 Rhombus Nanographenes

**Figure 14:**
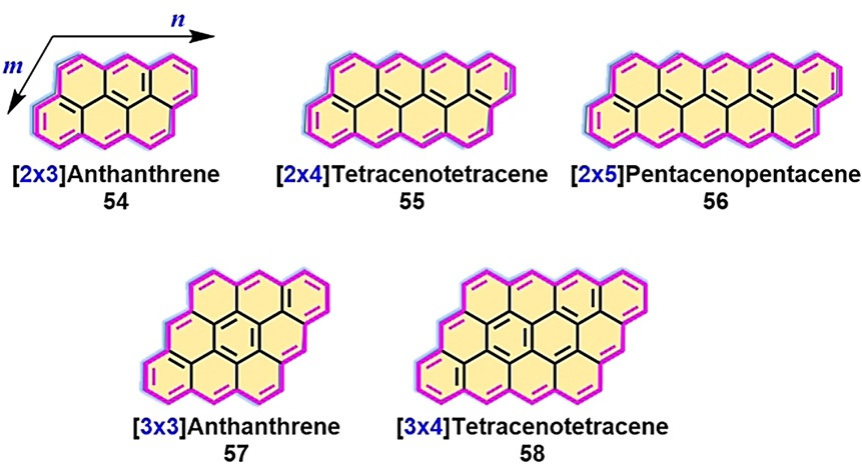
Chemical structures of the rhombus nanographenes.

**Scheme 7:**
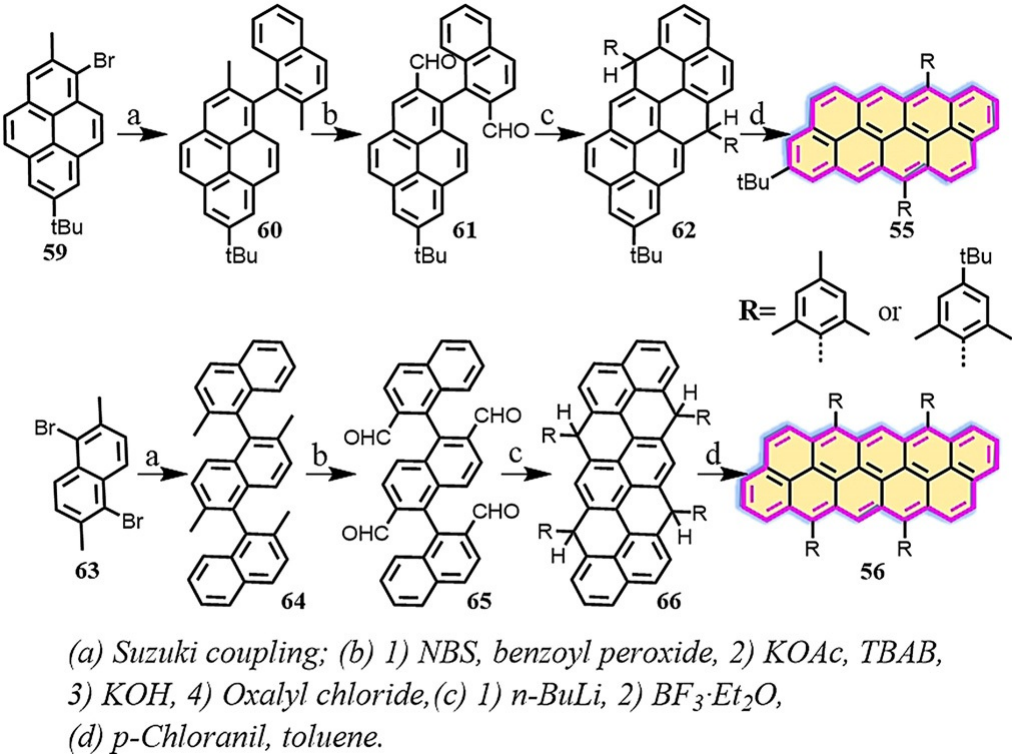
Synthesis of **55** and **56**.^[102]^

In contrast to the family of full zigzag‐edged triangulenes with intrinsic open‐shell character in the ground state, [*m*,*n*]rhombus NGs with full zigzag edges can be drawn with Kekulé structures, where *m* is the number of [*n*]acenes that annulate a rhombic nanographene (Figure 14). The [*m*,*n*]rhombus NGs reported thus far possess small diradical characteristics and display high chemical stability under ambient conditions. The [*m*,*n*]rhombus structures gain fewer Clar sextet rings in their diradical forms through resonance than the respective *peri*‐acenes and triangular‐shaped NGs. In 2012, Zhang and Briseno demonstrated the solution synthesis of [2×3]anthanthrene (**54**),[^100^, ^101^] which was investigated as an electron donor in organic solar cells and yielded a promising power conversion efficiency (PCE) of up to 2.0 %. Very recently, extended [*m*,*n*]*peri*‐acenoacenes such as [2×4]*peri*‐tetracenotetracene (**55**) and [2×5]*peri*‐pentacenopentacene (**56**) were successfully synthesized by Wu and co‐workers (Scheme 7).^[102]^

**Figure 15:**
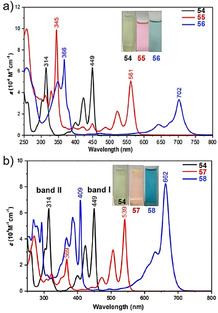
a) UV/Vis absorption spectra of **54**, **55**, and **56**.^[102]^ b) UV/Vis absorption spectra of **57** and **58**.^[103]^

The methyl groups in **60** and **64** were transformed into dialdehyde and tetraaldehyde groups by bromination, esterification, hydrolysis, and Swern oxidation to afford **61** and **65**. The dihydro/tetrahydro precursors **62**/**66** were synthesized by treatment of aldehydes **61**/**65** with phenyllithium followed by Friedel–Crafts cyclization. Dehydrogenation of **62**/**66** using *p*‐chloranil provided target compounds **55** and **56**. Single‐crystal analysis shows that both **55** and **56** have a flat π‐conjugated framework. Compared to [2×3]anthanthrene (**54**), the absorption maxima (*λ*_max_) of **55** and **56** are bathochromicaly shifted from *λ*=449 nm to 561 nm and 702 nm, respectively (Figure 15 a). Accordingly, compared to *peri*‐acenes, rhombic NGs **55** and **56** display somewhat larger band gaps and thus are much more stable under ambient conditions.

**Scheme 8:**
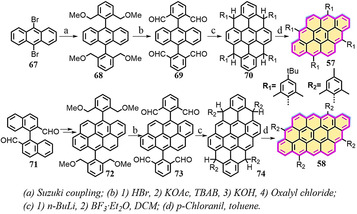
Synthesis of **57** and **58**.^[103]^

In addition, Wu and co‐workers recently reported the synthesis of extended [3×3]anthanthrene **57** and [3×4]tetracenotetracene **58** by extension of [2×3]anthanthrene along the lateral and longitudinal directions (Scheme 8).^[103]^ The synthesis of **57** and **58** is based on the vital intermediate tetraaldehydes **69** and **73**, in which anthracene and dibenzopyrene serve as the key building blocks, respectively. Both **57** and **58** display nearly planar π‐conjugated frameworks. Based on DFT calculations ((U)CAM‐B3LYP/6‐31G**), **57** has zero biradical character, whereas **58** possesses a small biradical contribution (*y*_0_=0.18). Similar to **55** and **56**, both **57** and **58** behave like closed‐shell compounds and feature intense absorptions at *λ*=539 nm and 662 nm, respectively (Figure 15 b). We also achieved the on‐surface synthesis of [3×3]anthanthrene‐based polymers, namely, poly(*para*‐dibenzo[*bc*,*kl*]coronenylene), which could be laterally fused to form zigzag‐edge‐extended GNRs.^[104]^

Compared to the above‐reported closed‐shell [*m*,*n*]rhombus NGs, an open‐shell singlet ground state will emerge when *m*,*n*≥5. Agapito et al. predicted that the [5,5]rhombus NG exhibits unique magnetic states that could be selectively tuned with the gate voltage.^[105]^ Thus, the bottom‐up synthesis of next‐generation rhombus NGs with full zigzag edges is expected to provide exotic low‐dimensional quantum phases of matter, such as magnetic exchange coupling behavior, in purely organic systems.

## 5 Heteroatom‐Doped Nanographenes

**Figure 16:**
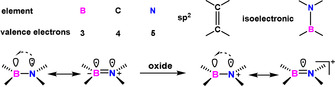
Isoelectronic structure of B‐N and C=C units.

In addition to edge topologies, the employment of heteroatoms in sp^2^‐carbon frameworks is another method to tune the intrinsic chemical and physical properties of PAHs.[^106^, ^107^, ^108^] The implementation of heteroatoms, such as an isoelectronic B‐N unit (Figure 16), has a significant influence on the electronic structures of PAHs.[^109^, ^110^] Moreover, compared with the nonpolar C=C bond, the B−N bond can be regarded as a zwitterionic double bond in its neutral state. The redox behavior of the B‐N unit has received significant interest recently (Figure 16).^[111]^

**Scheme 9:**
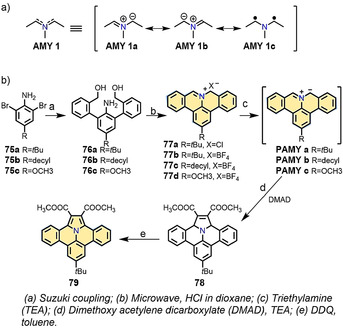
a) The resonance structures of **AMY 1**. b) Synthesis of **79**.^[116]^

Azomethine ylides (**AMY 1**; Scheme 9 a) are classic 1,3‐dipolar molecules.[^112^, ^113^] Interestingly, **AMY 1** possesses several resonance structures, since the negative charge on the allyl anion (**AMY 1 a** and **1 b**) can be distributed onto the neighboring carbon atoms. In addition to these two ionic structures, **AMY 1** also displays diradical character (**AMY 1 c**) in the ground state (Scheme 9 a).[^114^, ^115^] In 2014, we reported the synthesis of conjugated AMY‐containing aromatic rings (**PAMY**), in which the C‐N‐C unit is installed at the zigzag edge. This unprecedented **PAMY** can be used as a building block to synthesize nitrogen‐doped PAHs (N‐PAHs; Scheme 9 b).[^116^, ^117^] In general, the synthesis of zigzag‐edged **PAMY** consists of three steps. First, **76** is synthesized through the Suzuki coupling of **75** and 1‐hydroxy‐3*H*‐2,1‐benzoxaborole. Then, **77** with a zigzag edge is obtained by HCl‐induced cyclization of **76**. The final **PAMY** is synthesized by the treatment of **77** with basic conditions. Remarkably, zigzag‐edged **PAMY** enables the synthesis of extended N‐PAHs by 1,3‐dipolar cycloaddition. For example, **78** was directly synthesized through a 1,3‐dipolar cycloaddition with dimethoxyacetylene dicarboxylate (Scheme 9 b). Subsequently, the planar compound benzo[7,8]indolizino[6,5,4,3‐*def*]phenanthridine (**79**) was synthesized in 82 % yield by the oxidative dehydrogenation of **78**. Following this strategy, and by using different dipolarophiles, different kinds of unprecedented inner nitrogen‐doped NGs can be produced.[^117^, ^118^, ^119^, ^120^]

**Scheme 10:**
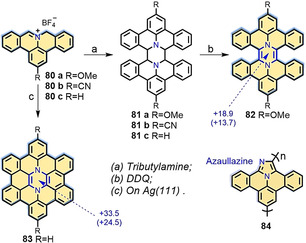
Solution‐based synthesis of **82**, on‐surface synthesis of N‐doped HBC **83**,^[121]^ and polyaromatic azaullazine chain **84**.^[122]^ Blue: NICS(1) value.

In addition to the cycloaddition reaction of **PAMY**, interestingly, **81** was produced through the dimerization of **80** in low yield (3 %; Scheme 10). The yield of **81** (51 %) could be greatly improved by increasing the temperature. Further oxidation of **81 a** with DDQ provided pyrazine‐incorporated hexabenzoperylene (HBP) **82**. However, **82** is extremely unstable, likely because of the antiaromatic nature of the pyrazine‐type core (Scheme 10). On the other hand, dimerized intermediate precursor **81 c** was not observed when **80 c** was annealed on the surface. Instead, diaza‐HBC **83** was directly formed (Scheme 10).^[121]^ Moreover, **80 b** substituted with a ‐CN group enabled the synthesis of polyaromatic azaullazine chain **84** (Scheme 10) on insulating layers, metal substrates, and in the solid state through intermolecular head‐to‐tail cycloaddition reactions.^[122]^

**Scheme 11:**
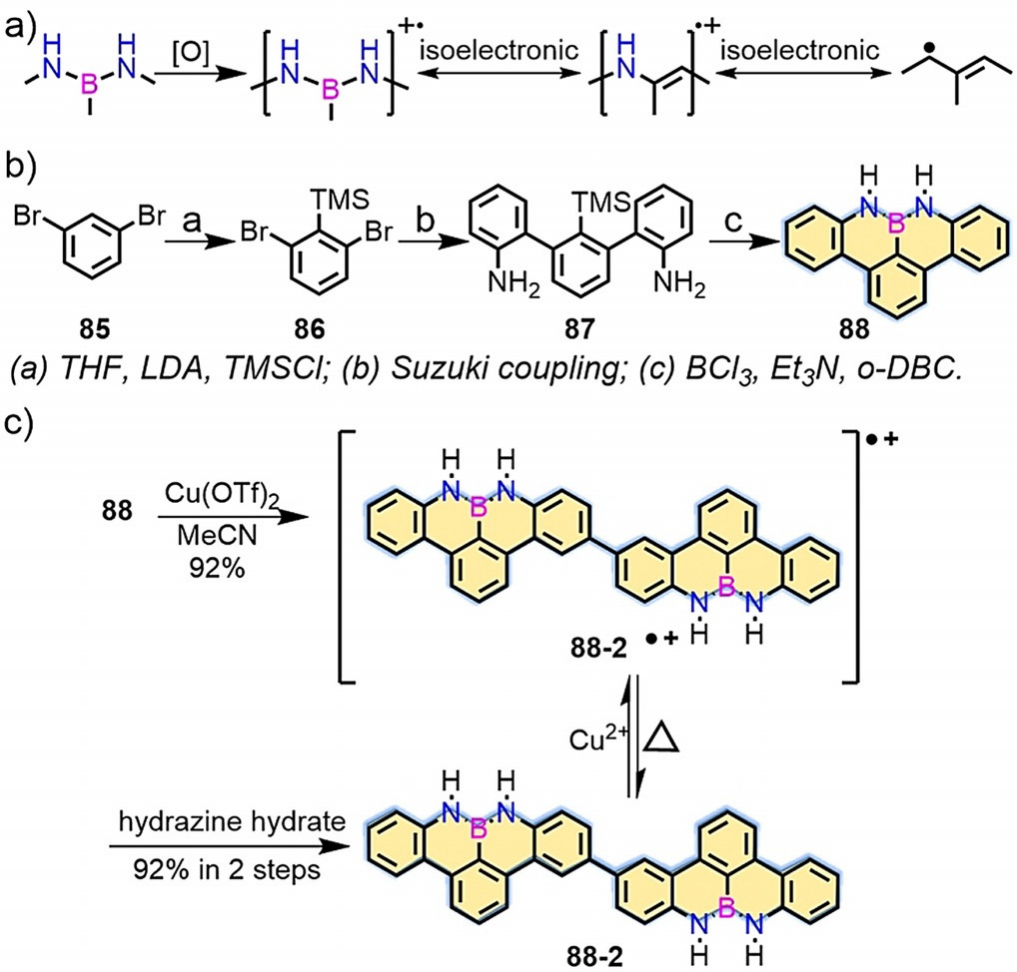
a) NBN‐edged structure and its radical cation and isoelectronic structures. b) Synthetic routes towards NBN‐doped **88**. c) The possible oxidation process of **88**.^[123]^

**Scheme 12:**
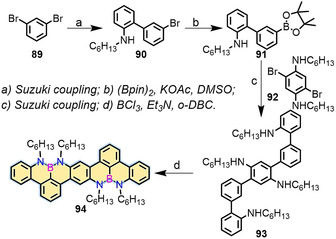
Synthesis of NBN‐doped **94**.^[123^

The introduction of a N‐B‐N motif at the zigzag edge not only affords the synthesis of stable zigzag‐edged NGs in solution but also provides the possibility to generate radical cations at the NBN‐doped edge through selective oxidation (Scheme 11 a).[^123^, ^124^, ^125^, ^126^] In 2016, our group demonstrated the first synthesis of 1,9‐diaza‐9*a*‐boraphenalenes containing NBN zigzagged edges (**88**; Scheme 11 b).^[123]^ NBN‐edged **88** was synthesized from **87** through electrophilic borylation, in which trimethylsilyl (TMS) served as the leaving group to form the NBN unit. Interestingly, **88** was dimerized to **88‐2** through chemical oxidation (Scheme 11 c). In addition, π‐extended dimer **94** was produced (Scheme 12), thus highlighting the potential of making GNRs containing NBN zigzag edges.

**Scheme 13:**
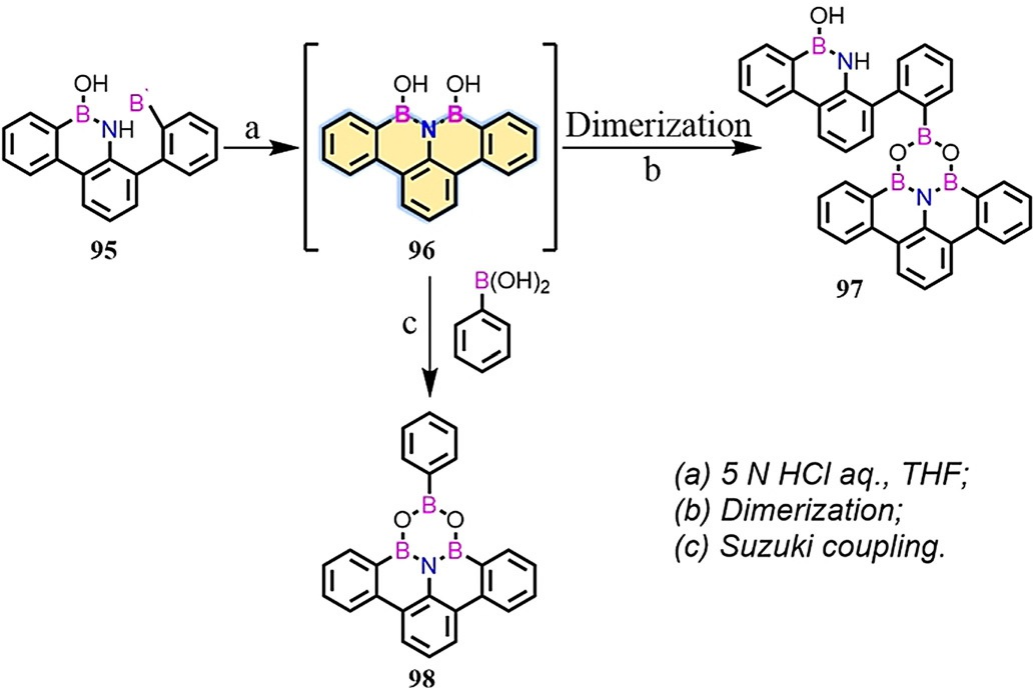
Synthesis of six‐membered B_3_NO_2_ heterocycles **97** and **98**.^[124]^.

**Scheme 14:**
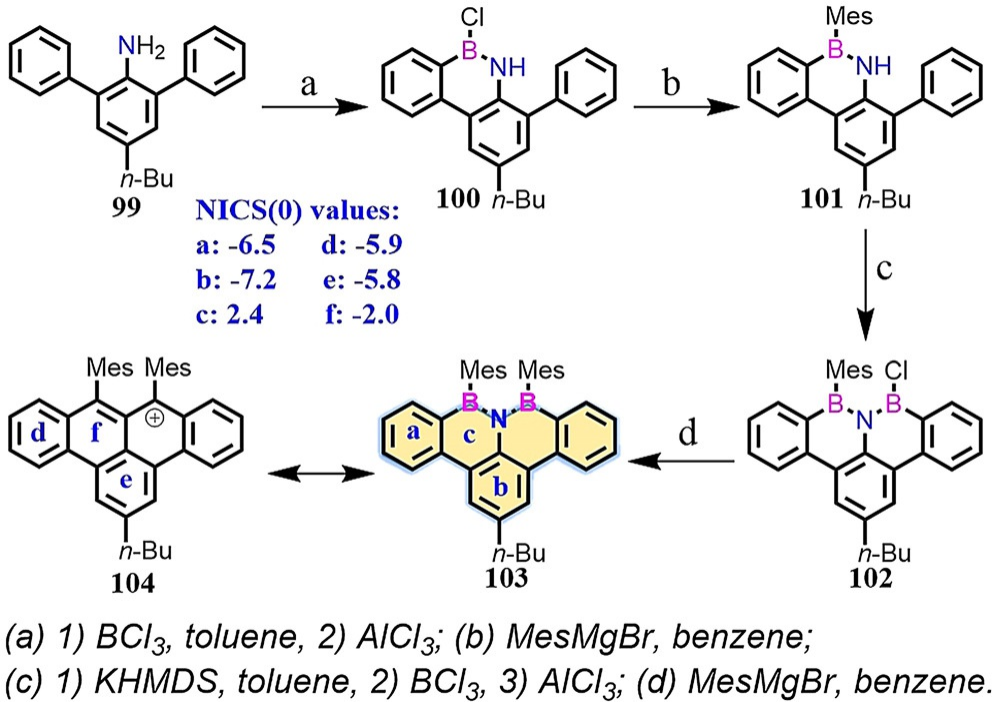
Synthesis of BNB‐edged **103** with mesityl groups.^[126]^

In contrast to BN‐ or NBN‐doped isoelectronic compounds, the B‐N‐B unit serves as an isoelectronic structure to its corresponding all‐carbon cation. In 2017, BNB‐edged benzo[*fg*]tetracene **96** was synthesized as an unstable intermediate, which could further react with itself (→**97**) or boronic acid (→**98**) to afford the B_3_NO_2_ ring (Scheme 13).^[124]^ In the same year, Bettinger and co‐workers demonstrated the synthesis of such compounds, which were stabilized by mesityl groups (**103**; Scheme 14).^[126]^ Moreover, BNB‐edged **103** is isoelectronic to its all‐carbon cation **104** (Scheme 14)^[127]^ with similar NICS values, thus indicating **104** and **103** possess similar aromaticity. In addition to these efforts, Zeng and co‐workers reported the synthesis of BNB‐embedded phenalene, which is isoelectronic with its all‐carbon phenalenyl cation.^[128]^

## 6 Zigzag‐Edged GNRs

**Scheme 15:**
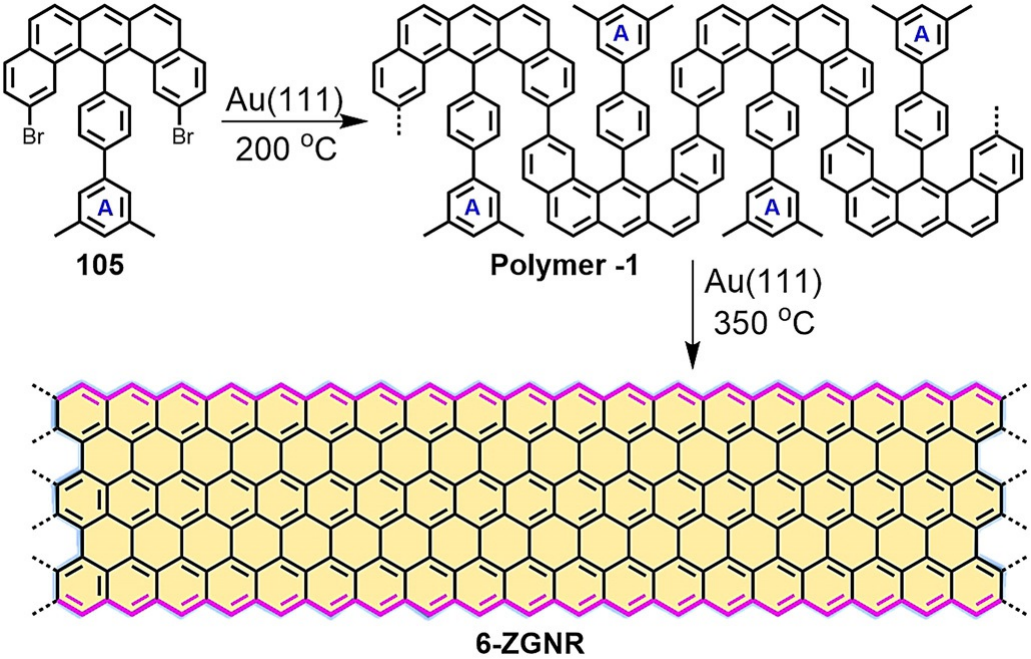
The synthetic route towards **6‐ZGNR** from the U‐shaped monomer **105** with two preinstalled methyl groups.^[69]^

The procedures established for the synthesis of PAHs can be further explored to construct GNRs from the corresponding polyphenylene polymers. In contrast to armchair‐edged GNRs (AGNRs) that display semiconducting behavior, zigzag‐edged GNRs (ZGNRs) demonstrate unique electronic and magnetic properties, including narrow band gaps and localized edge states.[^129^, ^130^] Therefore, although the solution‐based synthesis of AGNRs has been successfully demonstrated,[^131^, ^132^, ^133^] GNRs with rich zigzag edges have been limited thus far to on‐surface synthesis under UHV conditions because of their poor chemical stability. In 2016, the first bottom‐up synthesis of a full zigzag‐edged GNR (**6‐ZGNR**) on a Au(111) surface was realized by Fasel, Müllen, and us (Scheme 15).^[69]^ This method relied on the rational design of the U‐shaped dibenzoanthracene‐based precursor **105**, which allows on‐surface polymerization to form a snake‐type polymer (**Polymer**‐**1**). Moreover, two additional methyl groups on phenyl ring A are preinstalled (Scheme 15), which are essential to bridge with neighboring phenyl rings to establish additional zigzag‐edged rings.

**Figure 17:**
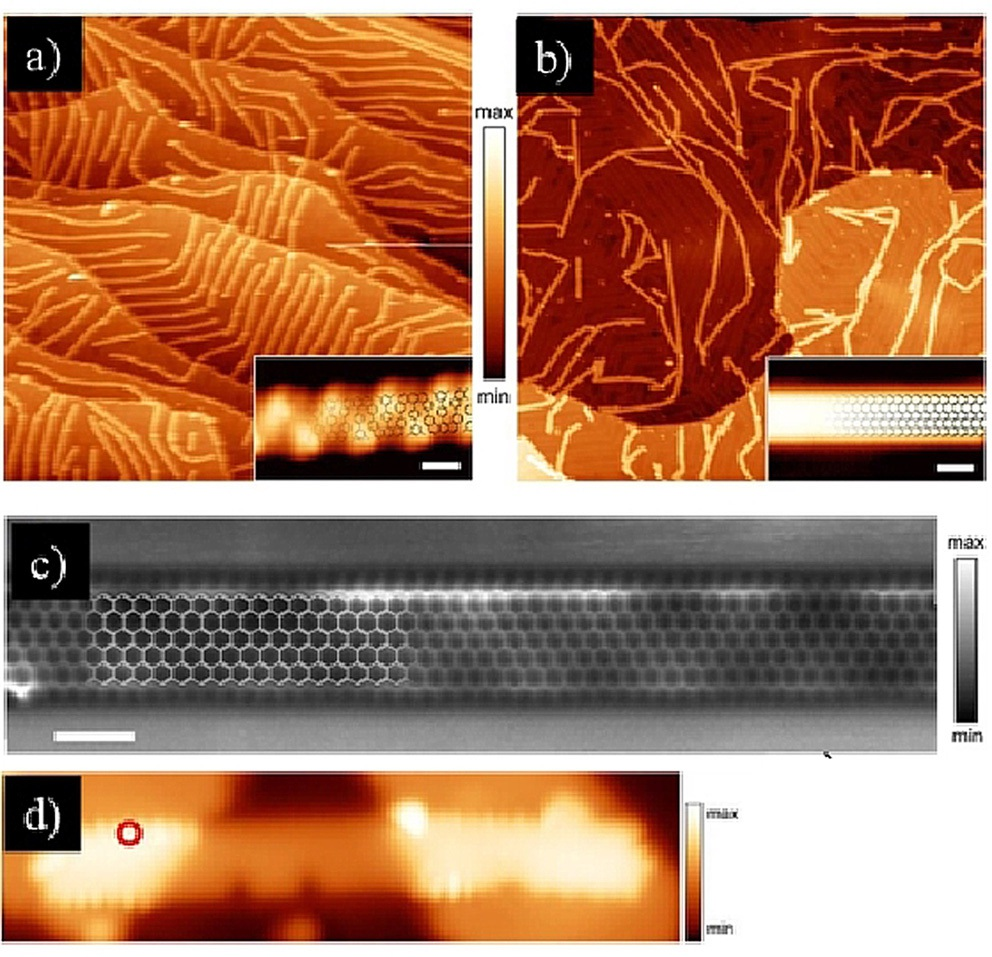
a) STM image of **Polymer**‐**1**. b) STM image of **6‐ZGNR**. c) nc‐AFM image of **6‐ZGNR**. d) STM image of **6‐ZGNR** on NaCl monolayer islands.^[69]^

For the on‐surface synthesis, monomer **105** was first sublimated at 150 °C. Then, **Polymer**‐**1** was synthesized by annealing at 200 °C (Figure 17 a). Finally, the fully zigzag‐edged **6‐ZGNR** was achieved by further annealing at 350 °C (Figure 17 b). Further structural details of **6‐ZGNR** could be unraveled by nc‐AFM (Figure 17 c), which demonstrated that its edge topology and width correspond to the conceived **6‐ZGNR**. However, the electronic edge states of zigzag edges are difficult to observe owing to the energetic electronic coupling between the gold surface and the ribbons. By manipulating **6‐ZGNR** with an STM tip onto insulating NaCl islands, clear evidence of the edge states could be observed as a result of electronic decoupling from the gold substrate (Figure 17 d).^[134]^ Edge states have also been detected on other carbon‐based nanostructures,[^135^, ^136^] such as graphene quantum dots (GQDs). The less‐defined zigzag edges resulted in their energy splitting being considerably smaller than that of **6‐ZGNR**. These results show that the magnetic and electronic properties of graphene nanostructures are very sensitive to their interactions with the underlying metal substrate and their edge roughness.

**Figure 18:**
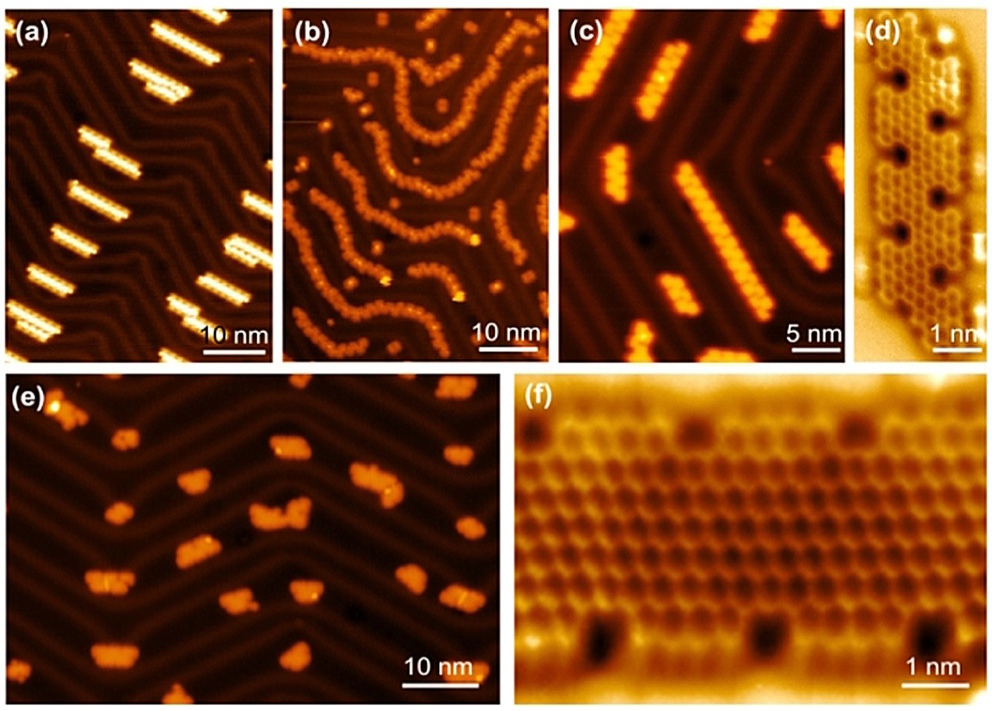
a) STM image of **106**. b) STM image of polymers **poly‐1**. c) STM image of **ZGNR1**. d) nc‐AFM image of **ZGNR1**. e) STM image of **ZGNR2**. f) nc‐AFM image of **ZGNR2**.^[137]^

**Scheme 16:**
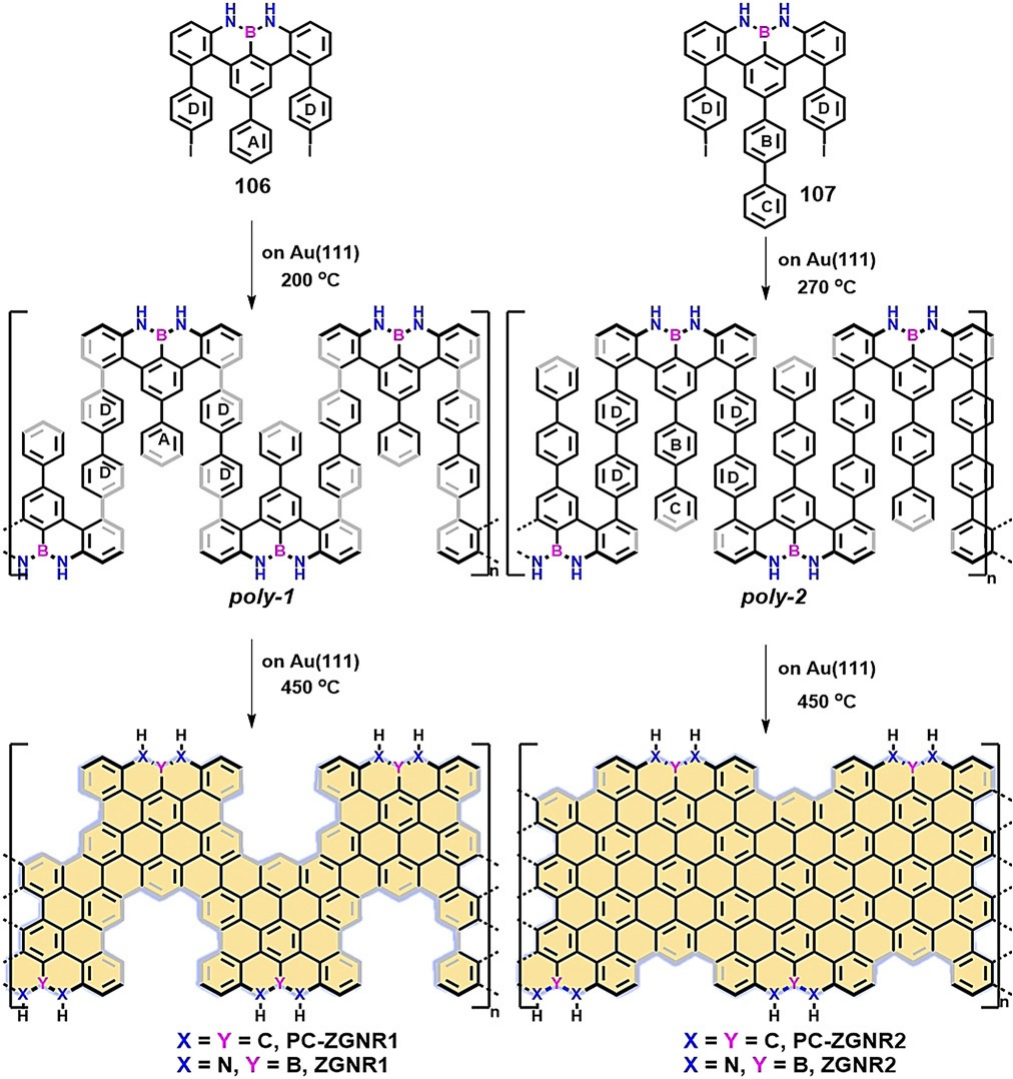
Synthetic strategy for NBN‐doped **ZGNR1** and **ZGNR2**.^[137]^

Following a similar synthetic strategy, the surface‐assisted synthesis of NBN‐edged ZGNRs (**ZGNR1** and **ZGNR2**; Scheme 16) from two U‐shaped NBN‐doped precursors (**106** and **107**) was recently reported by us.^[137]^ First, two iodo‐functionalized monomers **106** were synthesized by multistep organic synthesis, in which the NBN motif was preinstalled on the zigzag periphery. Then, monomer **106** was deposited onto the gold substrate (Figure 18 a). The swallow‐shaped polymer **poly‐1** was synthesized by annealing at 200 °C (Figure 18 b). Subsequently, the target **ZGNR1** could be obtained through intramolecular cyclodehydrogenation of **poly‐1** at 450 °C. The zigzag‐edge topologies were evidently unveiled through STM and nc‐AFM measurements (Figure 18 c,d). Monomer **107** containing one additional phenyl ring than **106** was further synthesized to afford **ZGNR2** (Figure 18 e,f), in which the zigzag‐edge proportion (57 %) was higher than that of **ZGNR1** (37 %). However, as a consequence of the substantial steric hindrance between the additional ring C with the side rings (such as rings D; Scheme 16) in polymer **poly‐2**, the length of the corresponding **ZGNR2** is shorter than that of **ZGNR1**. A comparison of the electronic structures of all‐carbon‐based ZGNRs (**PC‐ZGNR1**: 0.52 eV; **PC‐ZGNR2**: 0.27 eV; Scheme 16) shows the energy band gaps of **ZGNR1** (1.50 eV) and **ZGNR2** (0.90 eV) are much higher, which indicates that NBN doping plays a pivotal role in tailoring the electronic structures of graphene nanostructures. Since the NBN unit can be selectively oxidized to form the radical cation (Scheme 11 a), which corresponds to a pristine *C*_3_ carbon segment, this strategy offers further chemical modification for NBN‐doped ZGNRs.

**Scheme 17:**
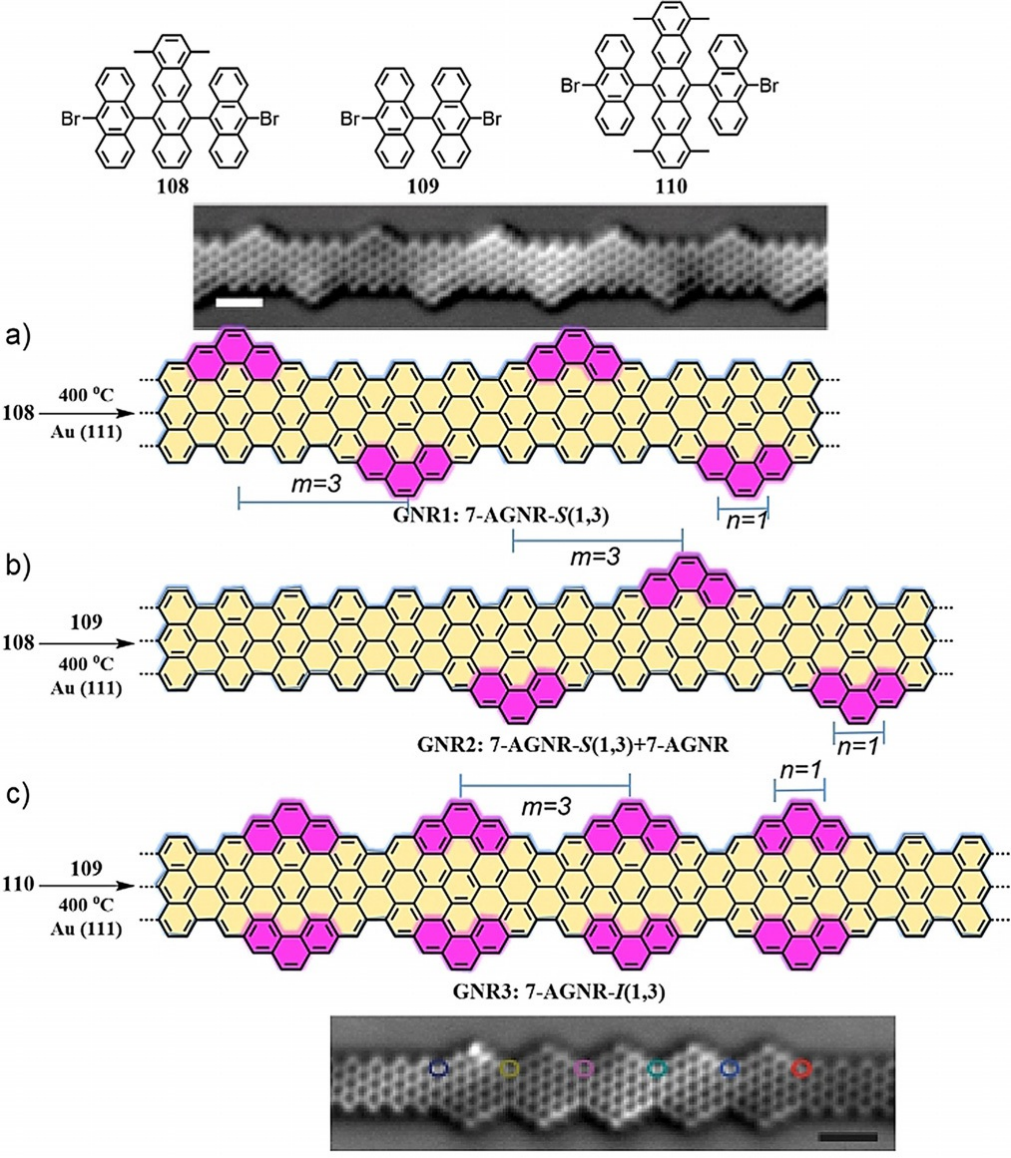
a) On‐surface synthesis of **GNR1**. b) **7‐AGNR**‐extended **GNR1**. c) **7‐AGNR**‐extended **GNR3**.^[139]^

Another significant breakthrough for GNRs was made in 2018 with the discovery of topological properties.[^138^, ^139^] It was shown that zigzag edges provide a platform to realize GNRs with exceptional physical properties. For example, topologically nontrivial (Z_2_=1) and trivial (Z_2_=0) GNRs were demonstrated by the introduction of short zigzag edge segments (pink segments) into *7*‐AGNR (Scheme 17).^[139]^ First, **GNR1** (***7*** **‐AGNR**‐*S*(1,3)) was synthesized from monomer **108** on a Au(111) surface (Scheme 17 a). The methyl groups play an essential role in the formation of the zigzag topology by bridging the neighboring rings. The chemical structure of **GNR1** was unambiguously confirmed using nc‐AFM (Scheme 17 a, inset). STS measurements showed the band gap of **GNR1** significantly decreased to 0.65 eV compared to pristine *7*‐AGNR (2.4 eV). Moreover, to determine whether **GNR1** behaves similarly to the topologically trivial class (Z_2_=0) or the nontrivial class (Z_2_=1), sequential sublimation of **108** (for **GNR1**) and **109** (for ***7*** **‐AGNR**) was carried out (Scheme 17 b). The experimental results demonstrated that the resulting **GNR1** could be classified as topologically trivial, with Z_2_=0, which is consistent with the tight‐binding prediction. In addition, by sequential sublimation of precursors **110** and **109, GNR3** (named ***7*** **‐AGNR**‐*I*(1,3)) and ***7*** **‐AGNR** could be produced (Scheme 17 c). The inset in Scheme 17 c displays the nc‐AFM image of **GNR3** (with 5 units), which is laterally expanded on both sides by ***7*** **‐AGNR** units. The d*I*/d*V* analysis shows that, in contrast to the trivial (Z_2_=0) **GNR1**, the resulting **GNR3** belongs to the topologically nontrivial class (Z_2_=1) and possesses topological end states.

## 7 Conclusions and Perspectives

This Minireview offers an overview of the recent developments in the synthesis of graphene nanostructures with dominant zigzag‐edged topologies. Both bottom‐up solution‐based and surface‐assisted synthetic strategies have been established and have achieved significant breakthroughs in the past few years. For example, *peri*‐tetracene with its biradical character in the ground state, which had been pursued for more than 70 years, was successfully synthesized in solution. π‐Extended triangulenes and full zigzag‐edged GNRs (**6**‐**ZGNR**) have been successfully synthesized by surface‐assisted routes. These atomically precise Z‐NGs and ZGNRs open up tremendous opportunities for the exploration and regulation of their fundamental physicochemical properties. A prominent example is the switching of the magnetic ground state from S=1/2 to S=0, which was recently reported for the concealed non‐Kekulé structure of Clar's goblet molecule, with quenched spins through atomic manipulation.^[99]^

Despite the incredible advances over the last few years, the development of Z‐NGs and ZGNRs is still in its infancy. Many challenges and opportunities remain for the synthetic exploration of this elusive type of graphene material, some of which are listed in this Minireview. Although the surface‐assisted synthesis of several prominent types of Z‐NGs and ZGNRs has been demonstrated, their solution‐based chemistry still lags behind because of their poor chemical stability. The introduction of bulky groups for kinetic protection or electron‐deficient groups (such as fluorine atoms) for thermodynamic stabilization can be a tradeoff strategy to obtain access to some stable Z‐NGs or ZGNRs in solution. Moreover, the introduction of nonplanarity by the incorporation of nonhexagonal rings into sp^2^‐carbon frameworks may provide an alternative pathway.[^140^, ^141^, ^142^, ^143^, ^144^] Only by overcoming the stability obstacle will further integration of these exotic materials in carbon‐based nanoelectronic devices become possible.

Heteroatom doping has been demonstrated as an efficient strategy for synthesizing stable Z‐NGs and ZGNRs with extended zigzag edges, which also enables chemical tuning of their electronic and magnetic properties. Therefore, further studies, both in solution and in on‐surface synthesis, focusing on the specific concentrations and positions of the heteroatoms in the zigzag‐edged graphene nanostructures will be essential for understanding the effects of substitutional doping on physicochemical properties. In addition to heteroatom doping, defect engineering, together with the structural design of zigzag edges, may provide an attractive method to tune the electronic and magnetic properties of Z‐NGs and ZGNRs.[^145^, ^146^] Our recent work shows that a five‐membered ring incorporated at the zigzag edge of nanographene can break the bipartite character of the sp^2^‐carbon lattice and induce a single net spin of S=1/2 .[^147^, ^148^] On the other hand, concealed non‐Kekulé NGs with intrinsic magnetism remain a class of less‐developed graphene nanostructures, and more effort will be needed for both solution and on‐surface synthesis. Moreover, given the high‐spin ground states, other attractive fundamental and technical prospects can be realized by the synthesis of one‐dimensional polymers, ribbons, and two‐dimensional networks by incorporating magnetic graphene molecules as building blocks.^[149]^ We hope that this Minireview will inspire new ideas in the design and synthesis of novel and stable zigzag‐edged graphene nanostructures, as well as the development of carbon‐based nanoelectronic devices.

## Conflict of interest

The authors declare no conflict of interest.

## Biographical Information

*Junzhi Liu received his PhD under the supervision of Prof. Klaus Müllen from the Max Planck Institute for Polymer Research (MPIP) in 2016. Then, he joined Prof. Xinliang Feng's group as a postdoctoral research associate at the Technische Universität Dresden (TU Dresden). In 2017 he became research group leader at the Chair for Molecular Functional Materials in TU Dresden. In August 2019, he started his independent research at the Department of Chemistry at The University of Hong Kong. His interests include the bottom‐up organic synthesis of topological carbon nanostructures and their applications in organic electronics*.

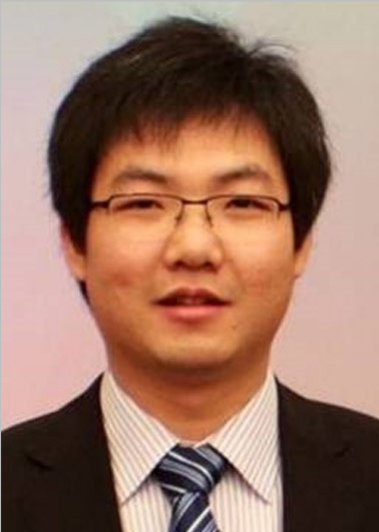

## Biographical Information

*Xinliang Feng has been full professor and the head of the Chair of Molecular Functional Materials at Technische Universität Dresden since 2014. His current scientific interests include organic synthesis, supramolecular chemistry of π‐conjugated systems, bottom‐up synthesis and top‐down fabrication of graphene and graphene nanoribbons, 2D polymers and supramolecular polymers, as well as 2D carbon‐rich conjugated polymers for (opto)electronic applications and materials for energy storage and conversion*.

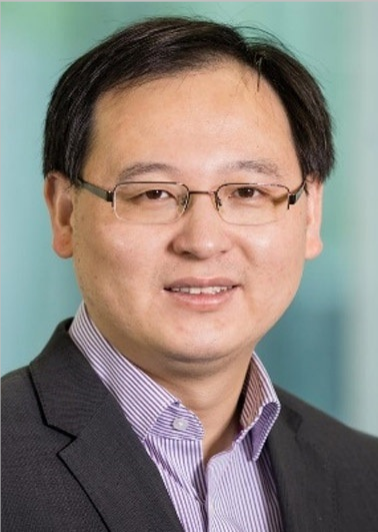

## Supporting information

As a service to our authors and readers, this journal provides supporting information supplied by the authors. Such materials are peer reviewed and may be re‐organized for online delivery, but are not copy‐edited or typeset. Technical support issues arising from supporting information (other than missing files) should be addressed to the authors.

Supplementary
