# Supplementary material for: Synthetic Tailoring of Graphene Nanostructures with Zigzag‐Edged Topologies: Progress and Perspectives
Source: Angew Chem Int Ed Engl. 2020 Oct 6;59(52):23386–401. doi: 10.1002/anie.202008838 (PMC7756885; doi:10.1002/anie.202008838)
Supplement: Supplementary file 1 — Supplementary [file ANIE-59-23386-s001.pdf]

Angewandte Chemie
Eine Zeitschrift der Gesellschaft Deutscher Chemiker

Supporting Information

Synthetic Tailoring of Graphene Nanostructures with Zigzag-Edged Topologies: Progress and Perspectives

Junzhi Liu and Xinliang Feng**

anie_202008838_sm_miscellaneous_information.pdf

There have been already nice review articles summarizing the acenes system, phenalenyl-based radicals and zethrene-type molecules, which also possess the zigzag-edge structures. Therefore, in order to avoid the overlap, in this Minireview, we will not particularly discuss these PAH molecules. For the reference, please find the followed literature:

Acenes system:

[1] W. Chen, F. Yu, Q. Xu, G. Zhou, Q. Zhang. "Recent progress in high linearly fused polycyclic conjugated hydrocarbons (PCHs, n > 6) with well-defined structures". *Adv. Sci.* **2020**, 7, 1903766.

[2] Q. Ye, C. Chi. "Recent highlights and perspectives on acene based molecules and materials". *Chem. Mater.* **2014**, 26, 14, 4046-4056.

Phenalenyl-based system:

[1] K. Ohashi, T. Kubo, T. Masui, K. Yamamoto, K. Nakasuji, T. Takui, Y. Kai, I. Murata. "4,8,12,16-Tetra-*tert*-butyl-*s*-indaceno[1,2,3-*cd*:5,6,7-*c'd'*]diphenalene: a four-stage amphoteric redox system". *J. Am. Chem. Soc.* **1998**, 120, 2018-2027.

[2] T. Kubo, A. Shimizu, M. Sakamoto, M. Uruichi, K. Yakushi, M. Nakano, D. Shiomi, K. Sato, T. Takui, Y. Morita, K. Nakasuji. "Synthesis, intermolecular interaction, and semiconductive behavior of a delocalized singlet biradical hydrocarbon". *Angew. Chem. Int. Ed.* **2005**, 44, 6564-6568.

[3] A. Shimizu, M. Uruichi, K. Yakushi, H. Matsuzaki, H. Okamoto, M. Nakano, Y. Hirao, K. Matsumoto, H. Kurata, T. Kubo. "Resonance balance shift in stacks of delocalized singlet biradicals". *Angew. Chem. Int. Ed.* **2009**, 48, 5482-5486.

[4] T. Kubo, A. Shimizu, M. Uruichi, K. Yakushi, M. Nakano, D. Shiomi, K. Sato, T. Takui, Y. Morita, K. Nakasuji. "Singlet biradical character of phenalenyl-based Kekulé hydrocarbon with naphthoquinoid structure". *Org. Lett.* **2007**, 9, 81-84.

[5] A. Shimizu, Y. Hirao, K. Matsumoto, H. Kurata, T. Kubo, M. Uruichi, K. Yakushi. "Aromaticity and π-bond covalency: prominent intermolecular covalent bonding interaction of a Kekulé hydrocarbon with very significant singlet biradical character". *Chem. Commun.* **2012**, 48, 5629-5631.

[6] A. Shimizu, T. Kubo, M. Uruichi, K. Yakushi, M. Nakano, D. Shiomi, K. Sato, T. Takui, Y. Hirao, K.Matsumoto, H. Kurata, Y. Morita, K. Nakasuji. "Alternating

covalent bonding interactions in a one-dimensional chain of a phenalenyl-based singlet biradical molecule having Kekulé structures". *J. Am. Chem. Soc.* **2010**, 132, 14421-14428.

[7] T. Kubo. "Phenalenyl-Based Open-Shell Polycyclic Aromatic Hydrocarbons". *Chem. Rec.* **2015**, 15, 218-232.

[8] T. Kubo. "Recent progress in quinoidal singlet biradical molecules". *Chem. Lett.* **2015**, 44, 111-122.

Zethrene-type molecules:

[1] E. Clar, K. F. Lang, H. Schulz-Kiesow. "Aromatische Kohlenwasserstoffe, LXX. Mitteil. 1): Zethren (1.12; 6.7-Dibenztetracen)". *Chem. Ber.* **1955**, 88, 1520-1527.

[2] H. A. Staab, A. Nissen, J. Ipaktschi. "Attempted preparation of 7,8,15,16-tetradehydrodinaphtho[1,8-*ab*; 1,8-*fg*]cyclodecene". *Angew. Chem. Int. Ed.* **1968**, 7, 226-226.

[3] R. H. Mitchell, F. Sondheimer. "The attempted synthesis of a dinaphth-1, 6-bisdehydro [10] annulene". *Tetrahedron* **1970**, 26, 2141-2150.

[4] H. A. Staab, J. Ipaktschi, A. Nissen. "Intramolekulare Wechselwirkungen zwischen Dreifachbindungen, VI Parallele Dreifachbindungen: Versuche zur Synthese des 7.8.15.16-Tetradehydro-cyclodeca[1.2.3-de: 6.7.8-d′e′]-dinaphthalins". *Chem. Ber.* **1971**, 104, 1182-1186.

[5] R. Umeda, D. Hibi, K. Miki, Y. Tobe. "Tetradehydrodinaphtho [10] annulene: a hitherto unknown dehydroannulene and a viable precursor to stable zethrene derivatives". *Org. Lett.* **2009**, 11, 4104-4106.

[6] R. Umeda, D. Hibi, K. Miki, Y. Tobe. *Pure Appl. Chem.* **2010**, 82, 871-878.

[7] T. C. Wu, C. H. Chen, D. Hibi, A. Shimizu, Y. Tobe, Y. T. Wu. "Synthesis, structure, and photophysical properties of dibenzo[*de*,*mn*]naphthacenes". *Angew. Chem. Int. Ed.* **2010**, 49, 7059-7062.

[8] L. Shan, Z. Liang, X. Xu, Q. Tang, Q. Miao. "Revisiting zethrene: synthesis, reactivity and semiconductor properties". *Chem. Sci.* **2013**, 4, 3294-3297.

[9] Y. Li, W. K. Heng, B. S. Lee, N. Aratani, J. L. Zafra, N. Bao, R. Lee, Y. M. Sung, Z. Sun, K. W. Huang, R. D. Webster, J. T. López Navarrete, D. H. Kim, A. Osuka,

J. Casado, J. Ding, J. Wu. “Kinetically Blocked Stable Heptazethrene and Octazethrene: Closed-Shell or Open-Shell in the Ground State?” *J. Am. Chem. Soc.* **2012**, 134, 14913-14922.

[10] W. Zeng, Z. Sun, T. S. Herng, T. P Gonçalves, T. Y Gopalakrishna, K. W. Huang, J. Ding, J. Wu. “Super-heptazethrene”. *Angew. Chem. Int. Ed.* **2016**, 55, 8615-8619.

[11] W. Zeng, T. Y. Gopalakrishna, H. Phan, T. Tanaka, T. S. Herng, J. Ding, A. Osuka, J. Wu. “Superoctazethrene: an open-shell graphene-like molecule possessing large diradical character but still with reasonable stability”. *J. Am. Chem. Soc.* **2018**, 140, 14054-14058.

[12] R. Huang, H. Phan, T. S. Herng, P. Hu, W. Zeng, S. Dong, S. Das, Y. Shen, J. Ding, D. Casanova, J. Wu. “Higher order π-conjugated polycyclic hydrocarbons with open-shell singlet ground state: nonazethrene versus nonacene”. *J. Am. Chem. Soc.* **2016**, 138, 10323-10330.
